# Retroelement—Linked Transcription Factor Binding Patterns Point to Quickly Developing Molecular Pathways in Human Evolution

**DOI:** 10.3390/cells8020130

**Published:** 2019-02-06

**Authors:** Daniil Nikitin, Andrew Garazha, Maxim Sorokin, Dmitry Penzar, Victor Tkachev, Alexander Markov, Nurshat Gaifullin, Pieter Borger, Alexander Poltorak, Anton Buzdin

**Affiliations:** 1 I.M. Sechenov First Moscow State Medical University, 119991 Moscow, Russia; danya.nikitin.orel@gmail.com (D.N.); sorokin@oncobox.com (M.S.); 2 Omicsway Corp., Walnut, CA 91798, USA; garazha@oncobox.com (A.G.); tkachev@oncobox.com (V.T.); 3 Faculty of Biology, Moscow State University, 119192 Moscow, Russia; markov_a@inbox.ru; 4 D. Rogachev Federal Research Center of Pediatric Hematology, Oncology and Immunology, 117198 Moscow, Russia; 5 Shemyakin-Ovchinnikov Institute of Bioorganic Chemistry, 117198 Moscow, Russia; 6 Faculty of Bioengineering and Bioinformatics, Lomonosov Moscow State University, 119992 Moscow, Russia; dmitrypenzar1996@gmail.com; 7 Faculty of Fundamental Medicine, Moscow State University, 119992 Moscow, Russia; gaifulin@rambler.ru; 8 Laboratory of the Swiss Hepato-Pancreato-Biliary (HPB) and Transplantation Center, Department of Surgery, University Hospital Zürich, Raemistrasse 100, CH-8091 Zürich, Switzerland; pieter.borger@usz.ch; 9 Program in Immunology, Sackler Graduate School, Tufts University, Boston, MA 02111, USA; alexander.poltorak@tufts.edu

**Keywords:** Human genome evolution, transcription factor, retrotransposons, molecular pathways, gene ontology, ChIP-seq, omics approach in genetics

## Abstract

**Background:** Retroelements (REs) are transposable elements occupying ~40% of the human genome that can regulate genes by providing transcription factor binding sites (TFBS). RE-linked TFBS profile can serve as a marker of gene transcriptional regulation evolution. This approach allows for interrogating the regulatory evolution of organisms with RE-rich genomes. We aimed to characterize the evolution of transcriptional regulation for human genes and molecular pathways using RE-linked TFBS accumulation as a metric. **Methods:** We characterized human genes and molecular pathways either enriched or deficient in RE-linked TFBS regulation. We used ENCODE database with mapped TFBS for 563 transcription factors in 13 human cell lines. For 24,389 genes and 3124 molecular pathways, we calculated the score of RE-linked TFBS regulation reflecting the regulatory evolution rate at the level of individual genes and molecular pathways. **Results:** The major groups enriched by RE regulation deal with gene regulation by microRNAs, olfaction, color vision, fertilization, cellular immune response, and amino acids and fatty acids metabolism and detoxication. The *deficient* groups were involved in translation, RNA transcription and processing, chromatin organization, and molecular signaling. **Conclusion:** We identified genes and molecular processes that have characteristics of especially high or low evolutionary rates at the level of RE-linked TFBS regulation in human lineage.

## 1. Background

**Figure 1:**
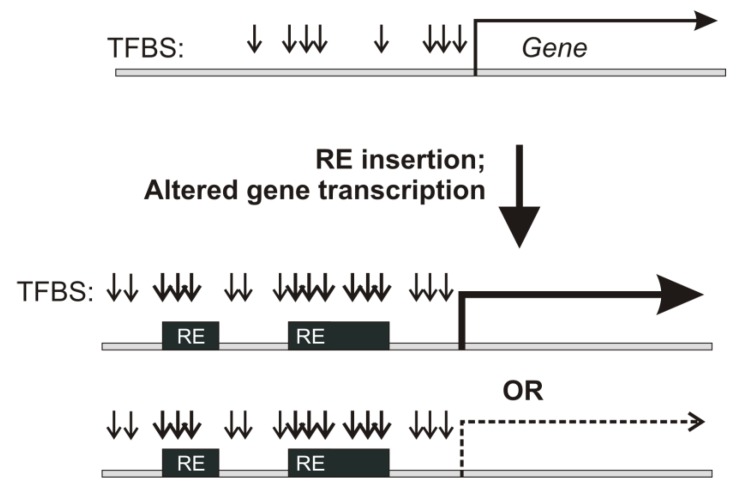
RE insertion in the proximity of transcription start sites can bring new TFBS and drastically alter gene expression.

Retroelements (REs) are transposable elements that occupy ~40% of the human genome [1,2] and regulate the expression of human genes by providing functional transcription factor binding sites (TFBS) [3,4,5,6], being one of the major forces of regulatory innovation [3,4,5]. RE inserts are far less conserved than the surrounding unique genomic regions [1]. RE-linked transcriptional regulation of human genes, therefore, can indicate quickly transforming gene regulatory modules [7,8,9] (Figure 1). 

The evolution of gene regulatory networks is one of the hot topics in current genetics [10,11]. At the level of individual genes, it can be investigated by analyzing structural and functional properties. Structural features include mutations or polymorphisms in gene regulatory regions [12] and inserts of REs [11,12]. Functional features may deal with the binding of transcriptional factors [13], DNA modification (e.g., methylation [14,15] or hydroxymethylation [16]) and chromatin rearrangement patterns [17,18], such as histone modifications [19].

Modern OMICS technologies enable direct interrogation of those features, e.g., screening mutations and polymorphisms by direct high throughput sequencing [20], or identifying TFBS, DNA and chromatin modifications by different versions of sequencing-followed immunoprecipitation techniques [21]. Bioinformatic approaches become key for the comprehensive analysis of “big data” generated by these methods [22,23,24,25]. 

A more profound level of data analysis may cope with groups of genes aggregated by some specific patterns, e.g. participation in molecular pathways [26,27], apparent common gene expression traits [28], or involvement in complex gene signatures [29]. Here, the approaches like *pathway activation strength* (PAS) calculation [30,31] or quantization of *Gene Ontology* (GO) clustering features [32,33] may be appropriate to assess oscillations of gene networks in response to any specific condition [34,35].

On the interface of studying both structural and functional features of gene evolution, a conceptually new method was recently published that analyzes the impact of RE-linked regulation for each gene [36]. It is based on a simple rationale to measure the proportion of gene regulatory items hosted by REs. Higher ratios are thought to indicate relatively quickly evolving regulatory modules, and vice versa. For every gene, a 10kb neighborhood of the transcriptional start site (TSS) was investigated, as TSS-proximal regions are thought to be enriched in functional regulatory modules, such as promoters and enhancers [37]. In its first application, proportions of RE-linked *TFBS* were calculated for all human genes based on the published experimental chromatin immunoprecipitation sequencing (ChIP-Seq) data [6] for hematological cancer cell line K562. ChIP-Seq makes it possible to precisely measure the binding of transcriptional factors with DNA, with greater number of sequencing reads (=hits) suggesting stronger binding with transcriptional factors at the same locus, and vice versa [38]. This approach of combining ChIP-Seq data and REs mapping was also for both types of data analysis, i.e. for the levels of individual genes and molecular pathways [36]. 

To assess the RE-associated regulatory charge for *individual genes*, a quantitative measure was introduced (*1*) termed *Gene RE-linked TFBS Enrichment score* (*GRE* score; Supplementary dataset 1). Conceptually, *GRE* for an individual gene is the sum of RE-specific TFBS hits mapped in a 10kb-neighborhood of its TSS, that is, normalized on the average content of RE-specific TFBS hits for all genes under study (Supplementary dataset 1). For every gene, *GRE* makes it possible to measure the regulation by RE-linked TFBS. However, this metric has the following limitation: Different genes have different regulation mechanisms and may have very different numbers of TFBS hits (both RE-linked and not) in their TSS neighborhood. The previous value (*GRE* score) shows if a gene is enriched or deficient by RE-linked TFBS hits relative to other genes, but the same gene may also be enriched in total (also non-RE) hits. 

It is important, therefore, to have a double-normalized value showing if gene regulation is specifically enriched in RE-linked hits relative to its total hits. To this end, a universal gene-specific metric (*2*) termed *Normalized Gene RE-linked TFBS Enrichment score* (*NGRE*) was proposed (Supplementary dataset 1), equal to the sum of RE-specific hits for a gene under investigation, double-normalized to the (i) average number of RE-specific hits for all genes and (ii) balanced total number of hits (not only RE-linked) for the same individual gene (formulas are given in Supplementary dataset 1) [36]. 

Similarly, at the level of *molecular pathways*, analogous additive values were proposed [36], termed *Pathway Involvement Index* (*PII*) and *Normalized Pathway Involvement Index* (*NPII*) (for formulas, see Supplementary dataset 1). *PII* is needed to assess the total impact of RE-linked TFBS on the regulation of an individual molecular pathway. The bigger *PII* suggests a higher impact of RE-linked hits on the overall regulation of a molecular pathway, and vice versa. However, *PII* is not informative to assess the importance of RE-linked regulation of a pathway in the context of its *total* regulation. To this end, the next metric termed *NPII* was proposed. The *NPII* (*Normalized PII*) is needed to estimate the *relative* RE-linked impact on the regulation of a whole molecular pathway. Higher *NPII* indicates a higher *relative* impact of RE-linked TFBS in the total regulation of a molecular pathway, and vice versa (Supplementary dataset 1) [36]. 

*NGRE* and *NPII* values were designed to estimate the relative impacts of REs in the regulation of individual genes and molecular pathways, respectively. Bigger NGRE and NPII evidence, respectively, a greater RE-linked regulatory impact for an individual gene or for a molecular pathway and, therefore, faster evolution of the corresponding gene regulatory network [36]. 

Several groups of molecular processes appeared highly *enriched* in the NGRE/NPII characteristics of their members. These dealt with the immunity and response to pathogens, negative transcriptional regulation, ubiquitination and proteasomal activity, extracellular matrix organization, regulation of STAT signaling, fatty acids metabolism, GTPase activity, protein targeting to Golgi, development, and functioning of perception and reproductive systems. In contrast, the most *deficient* in RE regulation processes related to the conservative pathways of embryo development [36]. However, these recent results were obtained for only one human cell line and could relate to the cell type- or tissue-specific patterns rather than to the evolutionary context of TFBS reshaping [39]. 

To distinguish cell type-specific and general evolutionary features, in this study we investigated into the distributions of RE-linked hits for all 13 human cell lines TFBS-profiled for 563 DNA-binding proteins during ENCODE project [40] and representing eight different tissues/organs from the different individuals (Supplementary file 2, Figure 2A). We found that 71% of totally mapped TFBS overlapped with the RE sequences, thus confirming RE’s status as the major source of TFBS for human cells. All the different cell lines investigated showed highly correlated patterns of NGRE distributions in pairwise comparisons, with correlation coefficients varying 0.5–0.95 with a median of ~0.85. The degree of RE-linked regulation for the individual genes was, therefore, roughly uniform in the different human tissues. We did the analysis on two evolutionary scales, first roughly corresponding to mammalian radiation and second to common human ancestry divergence with New World monkeys. The major processes enriched by integral RE-linked regulation dealt with olfaction, color vision, spermatogenesis and fertilization, particular aspects of immune and hormonal responses, intracellular molecular trafficking, amino acids, vitamins and fatty acids metabolism, xenobiotic metabolism, and detoxication. In contrast, the deficient pathways were involved in protein synthesis and ribosome biogenesis, RNA transcription and processing, nuclear chromatin organization, cell cycle, apoptosis, cell contacts, embryo development, most signaling pathways, cellular stress response, oxidative phosphorylation in mitochondria, and some other aspects of immunity. Among the top enriched cohort we found an approximate three times higher number of known genes for noncoding RNAs than in the bottom cohort of the same size. The pathway of gene silencing by microRNAs was also the top enriched pathway according to GO analysis.

## 2. Methods

### 2.1. Identification of RE-Specific Transcription Factor Binding Sites

Complete genome binding profiles of 563 investigated transcription factor proteins were extracted from the ENCODE database [41] for 13 human cell lines (K562, HepG2, HEK293, GM12878, MCF-7, A549, HeLa-s3, SK-N-SH, HCT116, Ishikawa, HEK293T, MCF-10A, GM12891) according to the standard ENCODE ChIP-seq protocol [6]. The reference human genome assembly 2009 (hg19) was indexed via Burrows–Wheeler algorithm using BWA software, version 0.7.10 [42]. Concatenation of fastq files with single-end or pairwise reads, alignment to the reference genome and filtering were done using BWA, Samtools (version 1.0), Picard (version 1.92), Bedtools (version 2.17.0) and Phantompeakqualtools (version 1.1) software [42] Aligned TFBS reads for each cell line were mapped on the RE sequences annotated by RepeatMasker (version 3.2.7) [43] and downloaded from the UCSC Browser [44] (RepeatMasker table). TFBS occurrence data were extracted from the bedGraph files [45] containing conservative IDR-thresholded peaks according to the standard ENCODE ChIP-seq analysis pipeline [46]. The folds change over control profiles for TFBS as well as the profiles for p-value to reject the null hypothesis that the signal at that location is present in the control were built using Macs software (version 2.1.0) [46] based on the alignment data. The list of transcription factors investigated here and raw ENCODE data files for each cell line is shown in Supplementary file 2.

Quality control of TFBS peaks analyzed in this study was performed using Irreproducible Discovery Rate correction according to ENCODE recommendations [46].

### 2.2. Evaluation of Evolutionary Age of Mapped REs

For each family of REs, average divergence from the consensus sequence was used as a measure of its evolutionary age. REs with an average divergence less than 8% were considered as *evolutionary younger fraction*, concerning the evolution of human lineage since its divergence from New World monkeys [47]. Another group contained all REs and roughly reflected genome shaping by REs since the origin of major eutherian clades [47]. Gene and molecular pathway enrichment by RE-linked TFBS was calculated separately for *all* and *young* REs. Average divergence from the consensus sequence was calculated using Repeatmasker software.

### 2.3. Measuring Gene Enrichment by RE–linked TFBS

The coordinates of human protein-coding genes were downloaded from the USCS Browser [44] (RefGenes table, genome assembly hg19). For each gene and cell line, all individual REs overlapping with the 10 kb-long neighborhood of its reference transcription start site were selected for further analysis. The 10-kb neighborhood covered an interval starting 5 kb upstream and ending 5 kb downstream the transcription start site. For every known gene in every cell line, we calculated its *GRE* and *NGRE* scores according to the formulas shown in Supplementary dataset 1. In total, we calculated *GRE* and *NGRE* scores for 24 389 human genes. 

### 2.4. Measuring Molecular Pathway Enrichment by RE–Linked TFBS

Gene architecture data of the molecular pathways were extracted from the following databases: BioCarta [48] (downloaded on March 2015), KEGG [49] (downloaded on June 2015), NCI [50] (downloaded on March 2015), Reactome [51] (downloaded on March 2015) and Pathway Central [52] (downloaded on March 2015). Data on molecular pathways structure were downloaded in .xml and .biopax formats from these databases and implemented in our computational algorithm [30]. In total, 3123 pathways were investigated. For each pathway in every cell line, we calculated *PII* and *NPII* scores according to the formulas shown in Supplementary dataset 1. 

### 2.5. Analysis of Cell Line Patterns of RE-Linked TFBS.

To screen for correlations of RE-linked TFBS distribution patterns among the cell lines under investigation, we first calculated Pearson correlation coefficients of gene-specific *GRE* values for each cell line under investigation except K562, with *GRE* values of previously investigated cell line K562 [36]. In this study, TFBS profiles for 260 TFs were studied for the K562 cell line. Second, we calculated all pairwise Pearson correlation coefficients of *GRE* values among all 13 cell lines under investigation (including K562). Similarly, the set of Pearson correlation coefficients was also calculated for the *NGRE* values as well. GRE and NGRE scores were calculated for the groups of *all* REs. Pairwise correlation for GRE and NGRE and the numbers of transcription factors investigated in each cell line are shown in Supplementary file 3. The entries in the main diagonal show numbers of transcription factors for each cell line; the intersections of the cell line vertical and horizontal rows above the main diagonal show Pearson correlation coefficients among the GRE scores for these two cell lines; and intersections of the cell line vertical and horizontal rows below the main diagonal show Pearson correlation coefficients among the NGRE scores. The same set of pairwise correlations for pathway scores of *all* REs, PII and NPII, are shown in Supplementary file 4. Similarly, the entries in the main diagonal show the numbers of transcription factors; intersections of the cell line vertical and horizontal rows above the main diagonal show Pearson correlation coefficients among the PII scores; and intersections of the cell line vertical and horizontal rows below the main diagonal show Pearson correlation coefficients among the NGRE scores. 

### 2.6. Gene Ontology Enrichment Analysis

Gene Ontology analysis of genes that are enriched or deficient in RE-linked TFBS regulation (RRE-enriched and RRE-deficient genes, respectively) was performed using DAVID (version 6.8) software [53] and Gorilla (version 1.0) software [54] using human genes IDs extracted from USCS Genome Browser [55]. The p-values specifying the significance of observed GO-terms enrichment were calculated using a modified Fisher’s exact test [56]. The cut-off for p-values was set as 0.05. The enrichment values of GO-terms and Annotation Clusters were calculated as fold changes of their occurrences in the sample and in the human genome [56].

### 2.7. Significance of Correlations 

The statistical significance of correlations was calculated as a Pearson correlation coefficient with a p-value using the Seaborn (version 0.9.0) package [57].

### 2.8. Significance of Gene Ontology Enrichment Analysis

To assess the confidence of the observed patterns for RE-impacted functional processes, we generated 500 sets of randomly permutated GRE and NGRE scores across the cell lines tested by randomly rearranging gene names. For each perturbation, we extracted a set of GRE-NGRE distribution-based 1219 top and bottom genes. These gene sets were profiled by DAVID software and top-100 GO terms were selected for each set by the lowest p-value for each random permutation. Finally, we compared the distributions of p-values for the top-100 GO terms for the permutated and real gene sets: real RRE-enriched and RRE-deficient genes were respectively compared with the distributions of RRE-enriched and RRE-deficient genes in random permutations. The overall data analysis pipeline is schematically depicted in Supplementary file 5.

All computational methods and all the codes used are freely available upon request to the authors.

## 3. Results

### 3.1. Mapping of RE-Specific TFBS

From the ENCODE project repository, we extracted experimental TFBS data based on the sequencing of immunoprecipitated DNA for 563 transcription factor proteins [6,58,59]. The sufficient amount of data including mapping TFBS for at least three transcription factors was available for 13 human cell lines (Supplementary file 2): myelogenous leukemia K562, transformed B cells GM12891, transformed lymphoblasts GM12878, cervix adenocarcinoma HeLa S3, endometrial adenocarcinoma Ishikawa, original and transformed human embryonal kidney HEK293 and HEK293T, breast cancer MCF-7 and MCF-10A, lung adenocarcinoma A549, hepatocyte carcinoma HepG2, colon carcinoma HCT116, and neuroblastoma SK-N-SH. These cell lines, therefore, represented eight cancerous or transformed human tissues/organs: blood, cervix, kidney, adrenal gland, mammary gland, lung, liver, and colon. Statistics for the TFBS data extracted and mapped on REs for these cell lines is shown in Table 1. 

In total, 277,187,723 TFBS hits could be mapped on the human genome for all these cell lines. Of them, 199,925,024 (72.1%) overlapped with the RE sequences, thus confirming that REs serve as the major source of TFBS in human cells. 

Considering previous reports, this proportion may seem high [60]. However, in our analysis, all multimapped TFBS reads were filtered out according to the standard ENCODE ChIP-seq mapping and filtering pipeline [42], so the results represented uniquely mapped TFBS reads. To confirm this TFBS proportion, we also validated the method used by parallel mapping of all human RE sequences extracted from USCS Genome Browser [55] on the human genome. In a good agreement with previously published data [1], REs mapped by the same approach occupied ~45% of human DNA, genome assembly hg19. We therefore found no technological drawbacks here and suggest that the proportion of 71% RE-linked TFBS is correct at least for the ENCODE primary cell culture datasets used.

However, this proportion was somewhat lower for the TFBS mapped in a 10-kb neighborhood of gene transcriptional start sites (TSS): among 43,042,026 totally mapped TFBS, only 61.4% overlapped with REs. This overall trend was representative for all cell lines under investigation (Table 2). The TSS-proximal TFBS hits were unevenly distributed among the major classes of REs: ~30% were attributed to SINEs; ~17%–to LINEs and ~7%–to LTR retrotransposons and endogenous retroviruses. For the total fraction of RE-linked TFBS hits (not only gene-proximal), these proportions were, respectively, 28%, 26% and 12%. 

These data evidence that TSS-proximal TFBS are peculiar because they have a ~1,2-fold lower proportion of RE-linked hits (Table 1). 

The distribution of TFBS hits for the different transcriptional factor proteins varied among the different cell lines (Supplementary file 6, color scale shows depth of TFBS mapping; blank spaces mean an absence of TFBS data for the respective transcriptional factor in the context of a given cell line in the ENCODE dataset). 

### 3.2. Calculation of Gene- and Pathway-Specific Characteristics of RE-Linked TFBS

For 25075 known individual human genes, we calculated the RE-linked TFBS absolute and normalized enrichment scores *GRE* and *NGRE*, respectively (Supplementary tables 1 and 7). For 3126 molecular pathways, we calculated the RE-linked TFBS absolute and normalized enrichment scores *PII* and *NPII*, respectively (Supplementary tables 1 and 8). All RE enrichment scores were calculated both for *all* and *evolutionary young* RE groups. The REs having a mean divergence from their consensus sequence of 8% and less were considered *evolutionary young*. This 8% divergence value roughly corresponds to the radiation of human ancestry from the New-World monkeys evolutionary clade [47]. It should be noted that for the most extensively profiled cell line K562, as much as 44% of genes were not impacted by *evolutionary young* REs and had zero GRE scores. To compare, for *all* Res, only 1,5% of human genes had zero GRE scores. A similar trend was seen in all the cell lines investigated here.

### 3.3. Comparison of RE-Linked TFBS Distribution Patterns among the Cell Lines 

**Figure 2:**
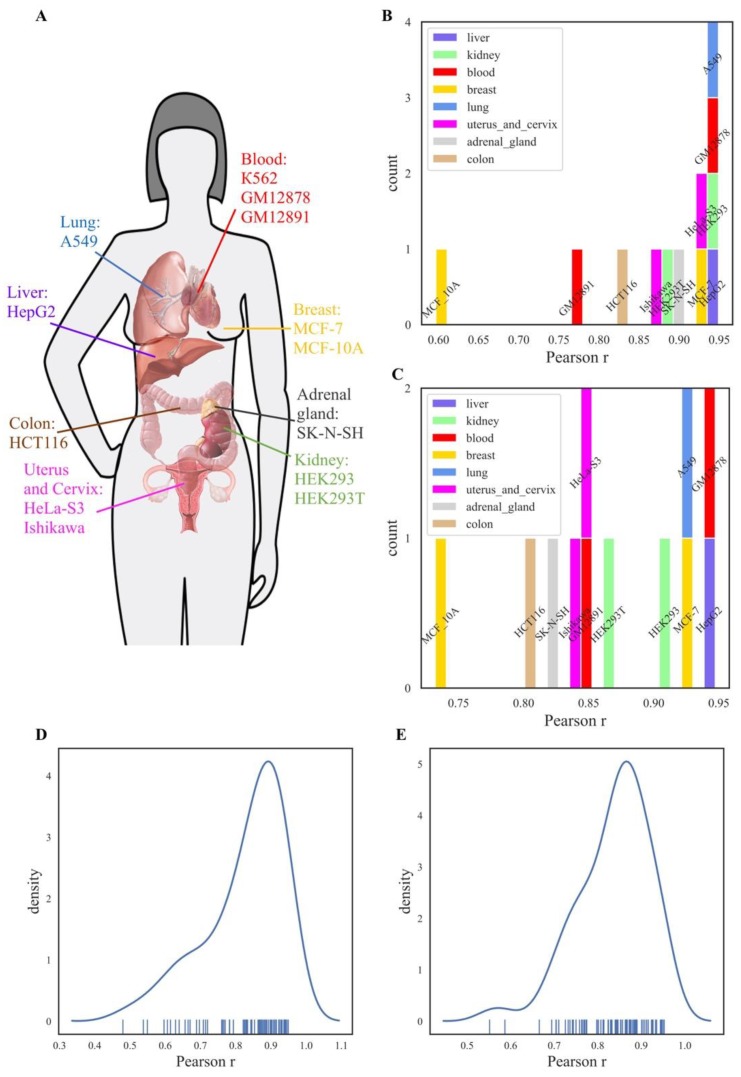
Comparison of *NGRE* and *NPII* scores between different cell lines for *all* REs. Colors denote human organs of cell lines origin. (**A**) Anatomical map of cell line origins investigated in this study. (**B**) Distribution of Pearson correlation coefficients of *NGRE* score of K562 cell line with *NGRE* scores of the 12 other cell lines investigated. (**C**) Distribution of Pearson correlation coefficients of *NPII* score of K562 cell line with *NGRE* scores of the 12 other cell lines. (**D**) Distribution of all pairwise Pearson correlation coefficients of *NGRE* scores of all 13 cell lines. (**E**) Distribution of all pairwise Pearson correlation coefficients of *NPII* scores of all 13 cell lines.

We next compared *NGRE* scores for all individual genes among the different cell lines investigated in this study (Figure 2). For comparisons with the previously published profiles of leukemia K562 cells [36], the *NGRE* scores were strongly correlated among the cell lines with a Pearson correlation r varying from 0.6 to 0.95 with a median value of ~0.9 (Figure 2B). Similarly, the molecular pathway-based *NPII* scores were also significantly correlated (r varying from 0.75 to 0.95 with median ~0.85), Figure 2C. *NGRE* and *NPII* correlations were calculated for *all* REs since RE-linked TFBS enrichment values were non-zero for the majority of genes. 

The correlations between K562 and other cell lines were not tissue-specific, as there were no tissue type-specific patterns observed in the distributions of the correlation coefficients (Figure 2A-C). For example, at the *NGRE* level, myelogenous leukemia cells K562 were equally strongly correlated (r~0.95) with the transformed lymphoblasts GM12878 cells and with the kidney HEK293, lung adenocarcinoma A549 and hepatocyte carcinoma HepG2 cells (Figure 2B). At the level of molecular pathways, the highest *NPII* score correlations with K562 were also seen for the same cell lines in addition to mammary gland carcinoma cells MCF-7 (Pearson r varying 0.9–0.95, Figure 2C). Finally, for all possible pairwise comparisons between all the cell lines investigated here, we observed unimodal distribution of Pearson correlations varying from ~0.5–0.95 with a median of 0.88, both at the gene and the pathway levels (Figure 2D and E, respectively). 

These findings strongly suggest that all 13 tested cell lines representing eight different human tissues are strongly congruent in RE-linked TFBS regulation of human genes and molecular pathways. 

### 3.4. Genes and Molecular Pathways Enriched or Deficient in all RE-Linked TFBS Regulation

**Figure 3:**
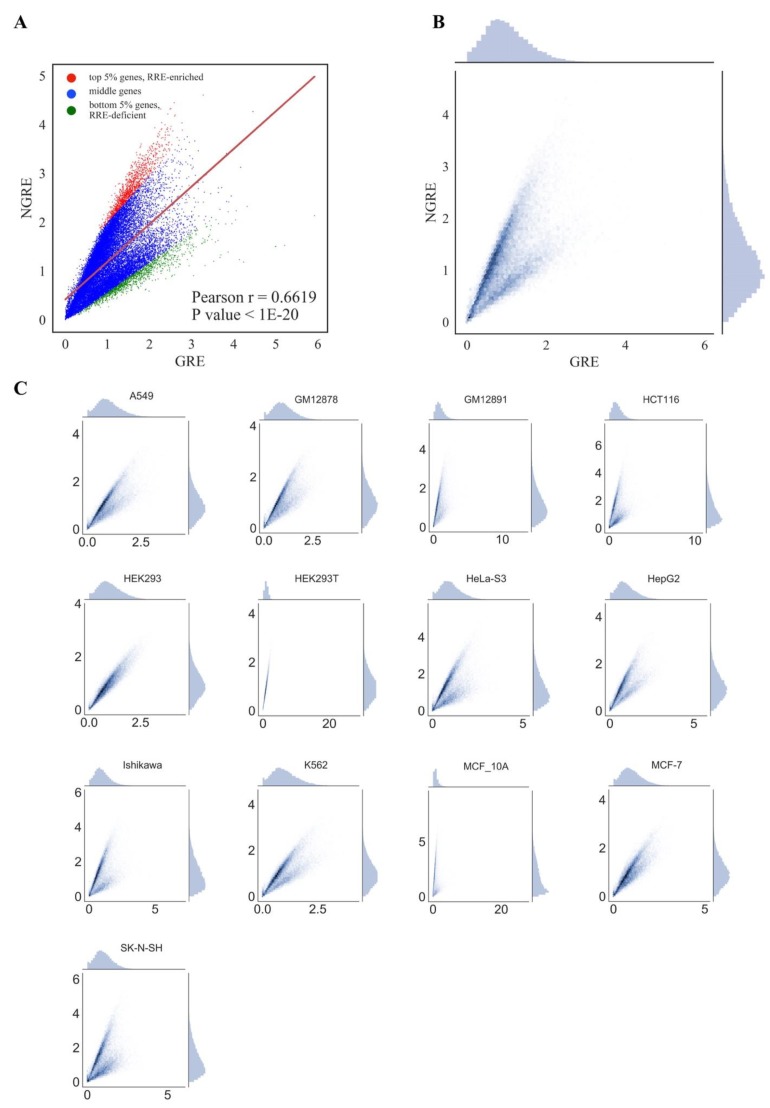
Comparison of *GRE* and *NGRE* scores across cell lines for *all* REs. (**A**) Comparison of mean GRE and mean NGRE scores. Each dot represents a single gene; *GRE* and *NGRE* scores were averaged across all cell lines. Genes enriched in RRE (regulation by retroelements) are shown as red dots. Genes deficient in RRE are shown as green dots. (**B**) Comparison of mean *GRE* and mean *NGRE* scores. Both scores were averaged across cell lines. Color depth is congruent with the number of single dots (each dot represents a single gene) in one grain. Univariate distributions of *GRE* and *NGRE* are shown in plot margins. (**C**) Comparison of *GRE* and *NGRE* scores for individual cell lines.

**Figure 4:**
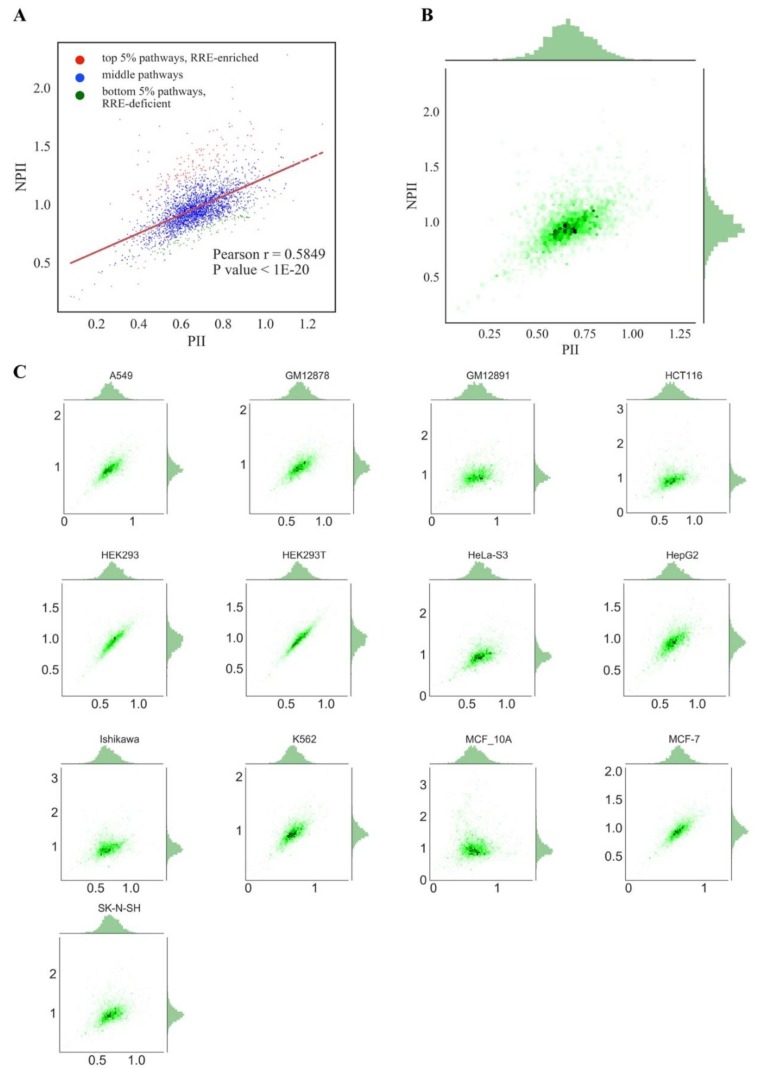
Comparison of *PII* and *NPII* scores across cell lines for *all* REs. (**A**) Comparison of mean *PII* and mean *NPII* scores. Each dot represents a single pathway; *PII* and *NPII* scores were averaged across all cell lines. Pathways enriched in RRE (regulation by retroelements) are shown as red dots. Pathways deficient in RRE are shown as green dots. (**B**) Comparison of mean *PII* and mean *NPII* scores. Both scores were averaged across cell lines. Color depth is proportional to the number of single dots (each dot represents a single pathway) in one grain. Univariate distributions of *PII* and *NPII* are shown in plot margins. (**C**) Comparison of *PII* and *NPII* scores for individual cell lines.

We next attempted to identify genes and molecular pathways enriched or deficient in RE-linked TFBS regulation (*RRE-enriched* and *RRE-deficient* genes/pathways). To this end, 24,389 human genes and 3123 pathways under investigation were examined respectively on scatter plots with an *abscissa* axis showing the *GRE* score for genes or *PII* for pathways, and, respectively, *ordinate* axis showing the *NGRE* score for genes and *NPII* for pathways (Figure 3 and Figure 4, respectively). This kind of presentation enables to visually distinguish genes/pathways that have a higher or lower *RRE* impact. Among the entries with the same *GRE* /*PII* scores, those having higher *NGRE* /*NPII* metrics will be enriched in *RRE*, end vice versa: Those with lower *NGRE* /*NPII* will be *RRE*-*deficient*. 

For both types of graphs, we observed very similar distribution trends among the different cell lines (Figure 3 and Figure 4). At the pathway level, *PII* and *NPII* scores were statistically significantly correlated for all the cell lines (Pearson r~0.58; Figure 4). Correlation was also strong at the gene level (*GRE* /*NGRE*), but in 12/13 of the cell lines tested we observed unusual yet very similar V-shaped distributions (Figure 3). This shape was remarkable and represented two rays of higher and lower slope coming from a zero point. The upper and lower rays accumulated, respectively, in relatively *RRE-enriched* and *RRE-deficient* genes.

We concluded, therefore, that different cell lines demonstrate highly similar patterns of RE-linked TFBS regulation at both the gene and pathway levels, as also suggested by the strong correlations among the cell lines (previous section). To operate with the universal values representing all cell lines tested, we next introduced the aggregated scores equal to the mean *GRE*, *NGRE, PII* and *NPII* values for all cell lines investigated, respectively (Figure 3A,B, Figure 4A,B). The distributions of the aggregated values were similar to those for the individual cell lines (Figure 3 and Figure 4).

To formalize the identification of the top *RRE*-*enriched* and *deficient* genes and pathways, we did the following. For the gene level, 1000 random sets each containing 500 genes were examined and a regression line (polynomial curve of first degree) was calculated using the Least Squares method [61]. Then two regression lines with the highest and lowest slope were selected. Then, 5% (1219) genes lying above the highest slope regression line with the maximal Euclidean distance to this regression line were considered as *RRE*-*enriched*. Similarly, 5% of the genes lying below the lowest slope regression line with maximal Euclidean distance from it were considered as *RRE*-*deficient* (Figure 3).

At the level of molecular pathways, a regression line (polynomial curve of first degree) was calculated using the Least Squares method [61] for the entire set of 3123 molecular pathways. Five percent (156) of pathways with maximal Euclidean distance lying above the regression line were considered as *RRE-enriched*, and 5% with maximal Euclidean distance lying below the regression line were considered as *RRE-deficient* pathways (Figure 4). We, therefore, identified 1219 top and 1219 bottom genes and 156 top and 156 bottom molecular pathways according to a measured aggregated RE-linked TFBS regulation in all13 human cell lines tested (Supplementary dataset 9 and 10).

### 3.5. Functional Characteristics of Top RRE-Enriched and Deficient Genes (all REs)

For the selected 1219 top and 1219 bottom genes, we performed Gene Ontology (GO) analysis using DAVID software [62,63] to identify if they are enriched by the clusters of genes included in any biological processes. The data on the top GO annotation clusters with a p-value < 0.05 are shown in Supplementary dataset 11. In total, 141 GO annotation terms were identified for the *RRE-enriched* genes, versus as much as 1022 (~7 times more) terms for the fraction of *RRE-deficient* genes. This finding may represent a phenomenon that more biological processes in human cells are evolutionary conserved than quickly evolving. 

We manually curated the identified annotation terms and could classify them into 27 major groups (Table 3). The significantly *RRE-enriched* groups of the processes dealt with the metabolism of amino acids, lipids and metals, detoxication and response to xenobiotics, with sensory perception, neurotransmission and fertilization. The *RRE-deficient* groups of processes were more numerous and featured protein translation, RNA transcription, intracellular signaling, cell adhesion and interaction, cell cycle progression, programmed cell death, metabolism of nucleic acids, carbohydrates, response to phorbol acetate, protein modifications, stress response and general virus response mechanisms, maintaining chromatin organization, electron transfer chain, and mitochondria functioning. 

Fifty annotation terms were linked with immunity; they were distributed differently depending on their functional roles. For example, immune cells migration/activation and cellular immune response by T- and NK cells were *RRE-enriched*, whereas B cells-related terms were *RRE-deficient* (Table 4).

**Figure 5:**
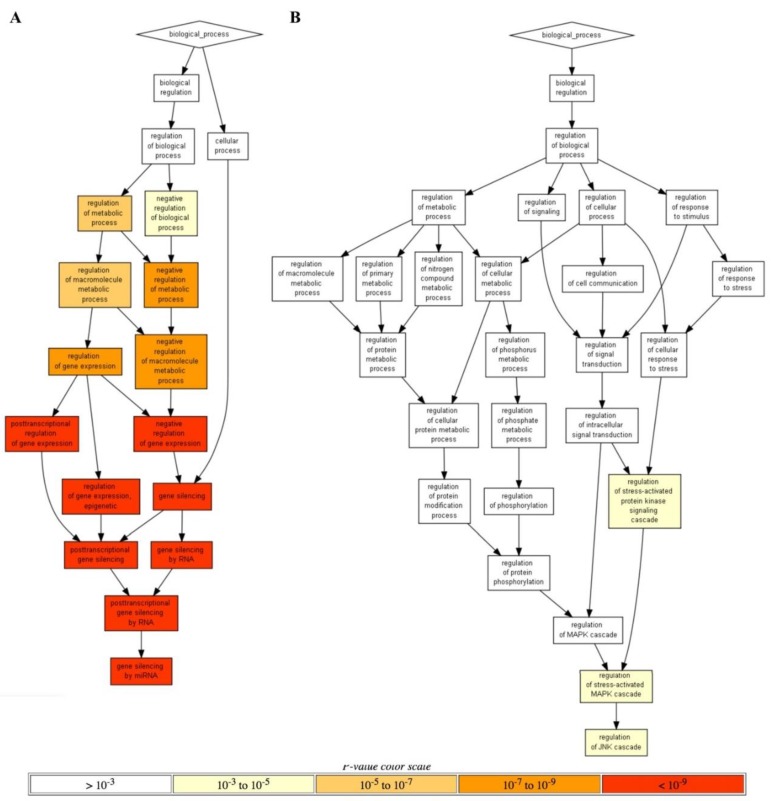
Hierarchically ordered GO annotated terms detected by Gorilla software for *RRE-enriched* (**A**) and RRE-deficient (**B**) genes for *all* REs.

In addition, another line of GO data analysis performed using Gorilla software [64] returned only the annotation terms that could pass significantly more stringent threshold. In this way, a unique yet extremely strongly statistically significant *RRE-enriched* annotation cluster (p < 10^−9^; Figure 5A) was for gene silencing by microRNAs, whereas the top two *RRE-deficient* processes were for the Regulation of stress-activated MAPK cascade and Regulation of JNK cascade (p < 10^−3^; Figure 5B). 

We next compared the microRNA (miR) contents of the *RRE-enriched* and *-deficient* gene sets (Table 5). The *enriched* group had 177 miR genes versus only 72 miR genes in the *deficient* group. Provided that the total content of miR genes in the human genome is 1865, a hypothesis can be accepted that the *RRE-enriched* group is also enriched in microRNA genes (p < 10^−17^) and that the *RRE-deficient* group is not enriched (p = 0.01). Similarly, the content of long non-coding RNA (lncRNA) genes also differed significantly among the gene sets (150 in the *enriched* versus only 18 in the *deficient* group; Table 5). With the total number of 1505 lncRNA genes in the human genome, this statistically supports the hypothesis that the *RRE-enriched* group is also enriched in lncRNA genes (p < 10^−16^) and that the *RRE-deficient* group is not enriched (p < 10^−15^). These findings clearly suggest that the regulation by RE-linked TFBS was particularly strongly recruited in the recent evolution of microRNA- and lncRNA-related mechanisms.

### 3.6. Characteristics of Top RRE-Enriched and Deficient Molecular Pathways (all REs)

We next examined the selected 156 top and 156 bottom molecular pathways sorted by RRE-enrichment as described above (shown in Supplementary file 10). As before, we manually curated the identified sets of molecular pathways and classified them into functional groups (Table 3). 

The *RRE-enriched* pathways were connected with the metabolism of amino acids, vitamins, lipids, sulfur and carbohydrates, molecular transport, response to and production of hormones, detoxication and response to xenobiotics, sensory perception and neurotransmission, and fertilization. The *RRE-deficient* pathways were related to protein translation, intracellular signaling including cell adhesion and interaction, cell cycle progression and programmed cell death, metabolism of nucleic acids, general virus response mechanisms, and chromatin organization. These functional groups, accordingly, showed a remarkable overlap of ~74% of the mentioned items for the comparisons at the gene and pathway levels (Table 3).

As before, many [4] differential *pathways* dealt with immunity, thus showing differential trends depending on their functional roles. Consistently, with the *gene* level of data analysis, cellular immune response by T- and NK cells were *RRE-enriched* (Table 4). Other *RRE-enriched* immune pathways regulated blood clotting, innate immunity and autoimmunity mechanisms. The *RRE-deficient* pathways operated with the general inflammation mechanisms and with the activation of antigen-presenting cells by T-helpers.

**Figure 6:**
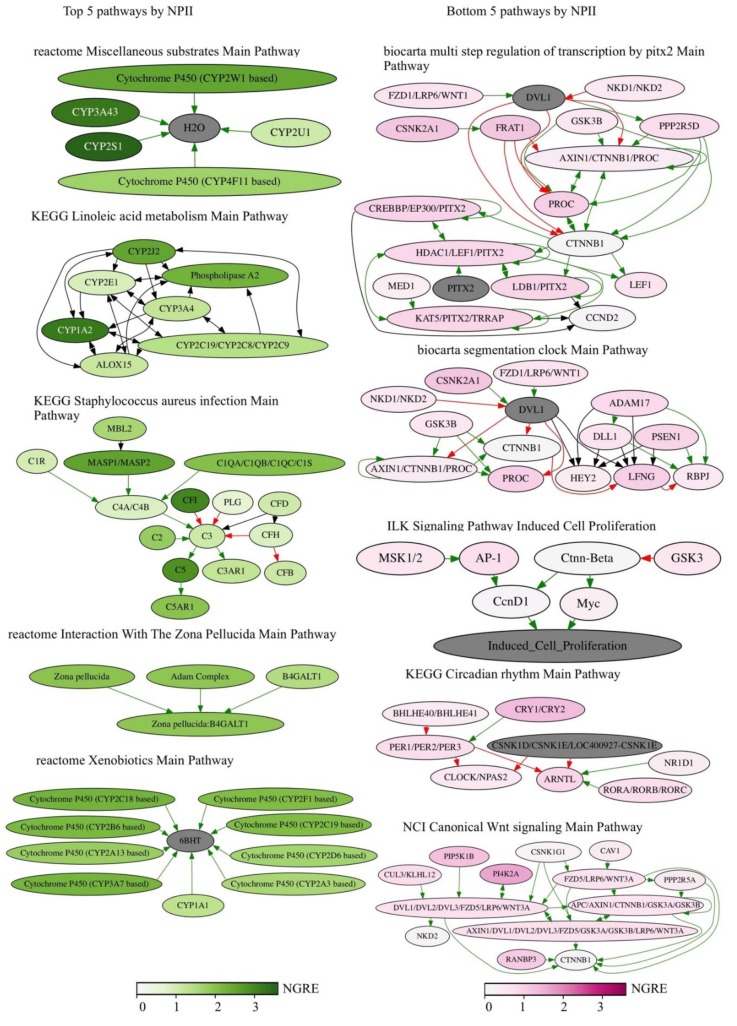
Top five *RRE-enriched* and *deficient* pathways sorted by *NPII* for *all* REs.

The top five *RRE-enriched* and -*deficient* pathways sorted by *NPII* representing the above processes are shown in Figure 6. 

### 3.7. Consensus Molecular Processes according to Gene- and Pathway- Based Assays (all REs)

Comparison of the results obtained at the gene and molecular pathway levels is outlined in Table 3. As mentioned above, the results obtained using these two alternative methods were highly congruent. The following molecular processes showed consensus regulation patterns using both types of data analysis. Processes with the *RRE-enriched* regulation: (1) posttranscriptional silencing by small RNAs; (2) DNA repair; metabolism of (3) amino acids and (4) lipids; (5) detoxication and metabolism of xenobiotics; (6) sensory perception and neurotransmission; (7) fertilization; (8) T- and NK-cellular immune response. 

More specifically, the *posttranscriptional silencing by small RNAs* (1) was represented by a single pathway and one GO term featuring gene regulation by microRNAs. Note also that according to GO analysis, the microRNA regulation was statistically the most strongly enriched cluster in the *RRE-enriched* subset. The content of microRNA and long non-coding (lnc) RNA genes in this subset was statistically also significantly enriched (p < 10^−16^). 

The *DNA repair* (2) cluster was represented by the Protein kinase pathway in nonhomologous end joining and by the GO terms of Mitotic recombination, DNA synthesis involved in DNA repair, Meiosis, and Strand displacement. 

The *metabolism of amino acids* (3) was represented by the pathways of D-arginine and D-ornithine metabolism, aspartate, asparagine, lysine, diphtamide, carnitine biosynthesis and cysteine, proline, hydroxyproline, beta-alanine, tryptophan and L-kynurenine catabolism, glutamate removal from folates, and conjugation of salicylate and benzoate with glycine. The GO terms were for the proline metabolic and tryptophan catabolic processes. 

The *metabolism of lipids* (4) had pathways of bile secretion, bile salt and organic anion SLC transporters, fatty acids cycling pathway, acyl-CoA hydrolysis, alpha-linolenic acid metabolism, alpha-oxidation of phytanate, beta-oxidation of unsaturated fatty acids, lipoxin biosynthesis, ether lipid metabolism pathway, synthesis of (16–20)–hydroxyeicosatetraenoic, epoxyeicosatrienoic and dihydroxyeicosatrienoic acids, and phosphatidylinositol acyl chain remodeling pathway. The GO terms were for lipid catabolic process, carboxylic ester hydrolase activity, arachidonic acid metabolism, epoxygenase and monooxygenase activity, and lipase activity.

The *detoxication and metabolism of xenobiotics* (5) cluster included pathways of CYP2E1 reactions, drug metabolism by cytochrome P450, nicotine, heme and bupropion degradation, caffeine metabolism, flavin-containing monooxygenase (FMO) oxidation of nucleophiles, formaldehyde oxidation, S-reticuline metabolism, aflatoxin activation and detoxification. The GO terms were for cellular response to xenobiotic stimulus, xenobiotic metabolic process and epoxygenase P450 pathway. 

The cluster of *sensory perception and neurotransmission* (6) processes has molecular pathways involved in olfactory signaling and transduction, visual signal perception via cones and GABA A (rho) receptor activation, dopamine receptors pathway and mechanism of acetaminophen activity, and toxicity pathway. The GO terms here were for the sensory perception of smell, odorant binding and olfactory receptor activity. 

The *fertilization* (7) was represented by a unique pathway of interaction with the zona pellucida and by several GO terms: fertilization, sperm-egg recognition, sperm flagellum, positive regulation of sperm motility, binding of sperm to zona pellucida, and single fertilization.

The *T- and NK-cellular immune response* (8) pathways regulated the phosphorylation of CD3 and T-cellular receptor zeta chains, downstream signaling in naive CD8 T-cells (alpha, beta T-cell proliferation), and CD28 co-stimulation in T-cell homeostasis. The GO terms were for the regulation of leukocyte-mediated cytotoxicity, natural killer cell-mediated immunity and its regulation.

Finally, the processes with the *RRE-deficient* regulation were: (9) nucleotide and DNA metabolism; (10) maintenance and modulation of chromatin structure; (11) protein translation and ribosome biogenesis; (12) intracellular signaling pathways; and (13) cellular mechanisms of antiviral response.

Here, the *nucleotide and DNA metabolism* (9) pathways controlled adenine and adenosine salvage, cleavage of the damaged purines, UMP biosynthesis, UDP-N-acetyl-D-galactosamine biosynthesis, GDP-L-fucose biosynthesis from GDP-D-mannose, and NADH repair. The GO terms were found for ATP metabolism, adenine transport, GTP binding, purine nucleosides and ribonucleotides metabolism, and pyrimidine binding.

The *maintenance and modulation of chromatin structure* (10) clade comprised pathways of PRC2 methylation of histones and DNA, HDACs histone deacetylation, arginine methyltransferases (RMTs) methylation of histone arginine residues, G2/M DNA damage checkpoint, and HDAC proteasomal degradation. The GO terms were found for the CENP-dependent centromere formation, for telomere formation and capping, lamin binding with chromatin, purine NTP-dependent helicase activity, histone exchange, histone lysine H3-K4 and H3-K9 methylation, acetylation and deacetylation, and for signal transduction in response to DNA damage.

The *protein translation and ribosome biogenesis* (11) pathways account for ribosomal and transfer RNA transcription, processing including RNA modifications such as wybutosine and 7-3-amino-3-carboxypropyl-wyosine biosynthesis, spliceosomal biogenesis and assembly, ribosomal assembly, tRNA aminoacetylation, ribosomal scanning, initiation, elongation and termination of translation for nonsense mediated decay. The identified GO terms fully functionally matched these molecular pathways.

The *intracellular signaling pathways* (12) formed the biggest group of molecular processes including 94 various pathways and 48 GO terms. These included all major aspects of human intracellular signaling (Table 3).

The *cellular mechanisms of antiviral response* (13) included pathways of host cell interaction with retroviruses, including APOBEC3-mediated resistance to HIV-1 infection. The GO terms were related to the assembly of viral capsids, viral transcription and translation, and IRES-dependent and cap-independent viral translational initiation.

**Figure 7:**
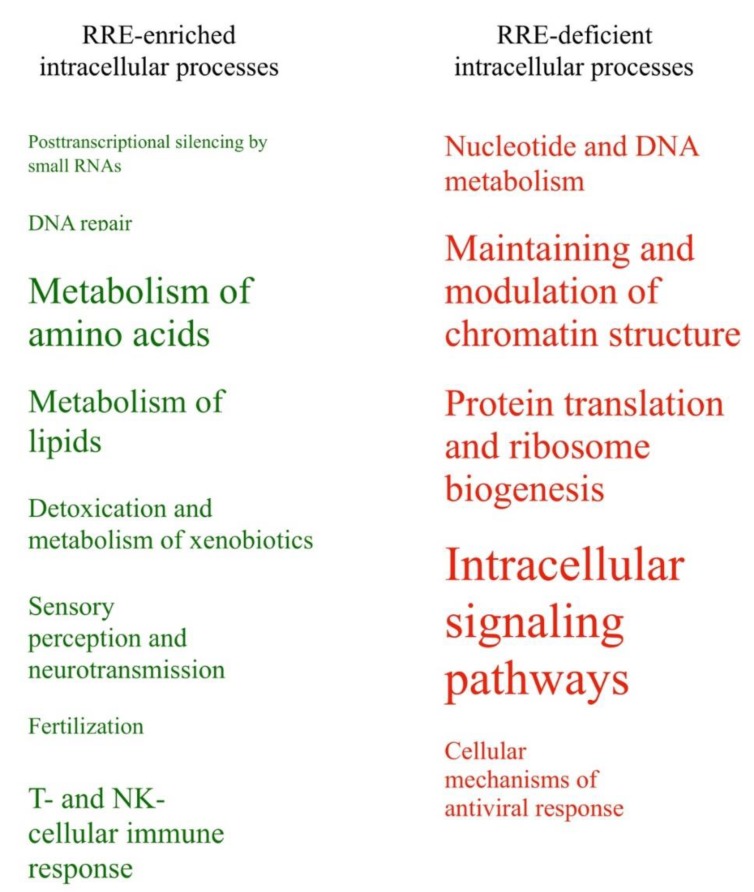
*RRE-enriched* and *RRE-deficient* molecular processes for *all* REs.

All these categories were represented by different numbers of enclosed pathways and GO terms (Table 3). Schematically, these processes linked with enriched or deficient RRE-regulation are shown in Figure 7 in relation with the number of enclosed features. Fourteen other groups of processes (50%) were identified as either RRE-enriched or deficient using only one of the methods used (either *gene*- or *pathway*-based), and only one group (~4% of the total amount of groups considered) for the metabolism of carbohydrates showed ambiguous trends in these two types of analyses (Table 3). 

### 3.8. Randomness Test of GRE and NGRE-Based Data

**Figure 8:**
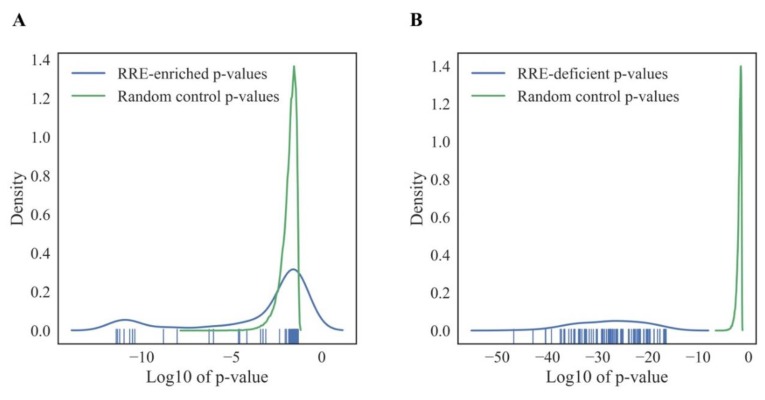
Random control of GO processes enrichment in *RRE-enriched* (**A**) and *RRE-deficient* (**B**) genes for *all* REs.

In order to assess the confidence of the observed patterns of RE-impacted intracellular processes, we generated 500 random permutations, averaged across cell lines, of GRE and NGRE value sets for all genes tested by randomly rearranging gene names. For each iteration, we created a GRE-NGRE scatter plot and extracted 1219 top and 1219 bottom genes as described above. Next, we analyzed these randomly generated gene sets using DAVID software, and top-100 GO terms were selected by the lowest p-value for each random permutation. Finally, we compared the distribution of p-values for the real and random gene sets (Figure 8A for RRE-enriched and Figure 8B for RRE-deficient genes). This randomness analysis showed that the RRE-deficient molecular processes identified here are nonrandom, because random and real distributions did not intersect (Figure 8B). Interestingly, a major part of RRE-enriched GO-terms overlapped with the random distribution (Figure 8A), although there was a specific peak of outstandingly non-random 10 GO terms among the RRE-enriched items, which had smaller p-values than each of the random items in 500 permutations. Since none of the 500 random permutations generated GO-terms with p-values lower than those observed for the real RRE-enriched or RRE-deficient genes, the overall q-values of confidence for both groups were smaller than 0.002, which indicates high confidence level of the molecular processes identified.

### 3.9. Functional Characteristics of Top RRE-Enriched and Deficient Genes (Evolutionary Young REs)

Similarly, we analyzed the top RRE-enriched and deficient molecular processes by using the fraction of *evolutionary young* human retrotransposons. We first identified the top 673 RRE-enriched and 673 RRE-deficient genes. A smaller number of top genes compared to *all* REs was taken because only 44% of genes had non-zero *GRE* scores and could be properly analyzed. For the genes selected, we performed GO analysis using DAVID software. The data on the top GO annotation clusters with a p-value of <0.05 are shown in Supplementary dataset 12. In total, 55 GO annotation terms were identified for the *RRE-enriched* genes, versus as much as 730 (~13 times more) terms for the fraction of *RRE-deficient* genes. As in the case of *all* REs, there were more evolutionary conserved than quickly evolving intracellular molecular processes identified.

We manually curated the identified annotation terms and classified them into 24 major groups (Table 6). Similarly to *all* REs, the significantly *RRE-enriched* groups were connected with sensory perception and neurotransmission, immune system, metabolism of lipids, detoxication, and response to xenobiotics. The *RRE-deficient* groups of processes were also generally in line with *all* REs and included protein translation, RNA transcription, intracellular signaling, cell adhesion and interaction, cell cycle progression, programmed cell death, metabolism of nucleic acids and carbohydrates, protein modifications, stress response and processes interfering with viral life cycle, maintaining chromatin organization, oxidative phosphorylation, and mitochondrial functioning. Immunity processes had contradictory trends and were represented by 15 annotation terms (Table 7).

**Figure 9:**
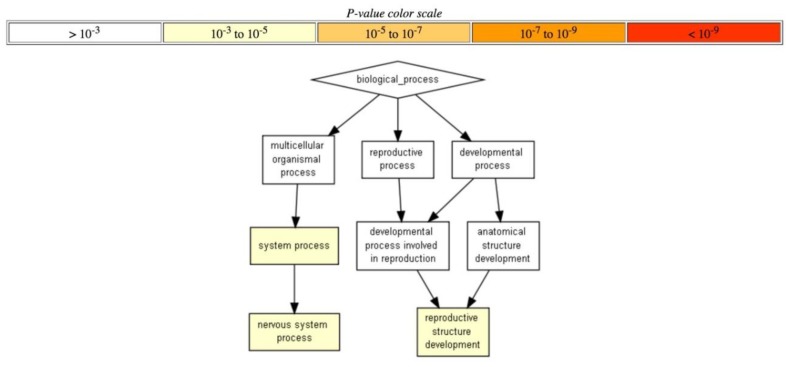
Hierarchically ordered GO annotated terms detected by Gorilla software for RRE-deficient genes for *evolutionary young* REs.

Alternative analysis using Gorilla software showed that only *RRE-deficient* genes were organized into a distinct network (processes of nervous system and reproductive organs development), whereas no statistically significant processes were found for RRE-enriched genes at the level of confidence p < 10^−3^. Figure 9). 

### 3.10. Characteristics of Top RRE-Enriched and Deficient Molecular Pathways (Evolutionary Young REs)

Results of analysis and manual curation (Table 6) of the top 152 RRE-enriched and 152 RRE-deficient molecular pathways for *evolutionary young* REs (shown in Supplementary file 13) were congruent with the results for *all* REs. 

The *RRE-enriched* pathways featured the metabolism of amino acids, polyamines, vitamins, lipids, sulfur and carbohydrates, molecular transport of small molecules, detoxication and response to xenobiotics, blood clotting, cell cycle progression, and cell death. The *RRE-deficient* pathways, in turn, related to protein translation and maturation, intracellular signaling including cell adhesion and interaction, cell cycle progression and programmed cell death, metabolism of nucleotides and nucleic bases, amino acids, polyamines, lipids, carbohydrates, and virus life cycle mechanisms. Overlap of processes at the level of genes (GO terms analysis) and pathways was ~50%, that is lower than in the case of *all* REs (Table 3, Table 6).

Unexpectedly, the same groups of immunity processes were identified for *RRE-enriched* and *deficient* pathways: those linked with innate immunity, T-cell mediated immunity and inflammation. In the first two groups, more pathways were RRE-enriched than RRE-deficient (Table 7).

### 3.11. Consensus Molecular Processes according to Gene- and Pathway- Based Assays (Evolutionary Young REs)

**Figure 10:**
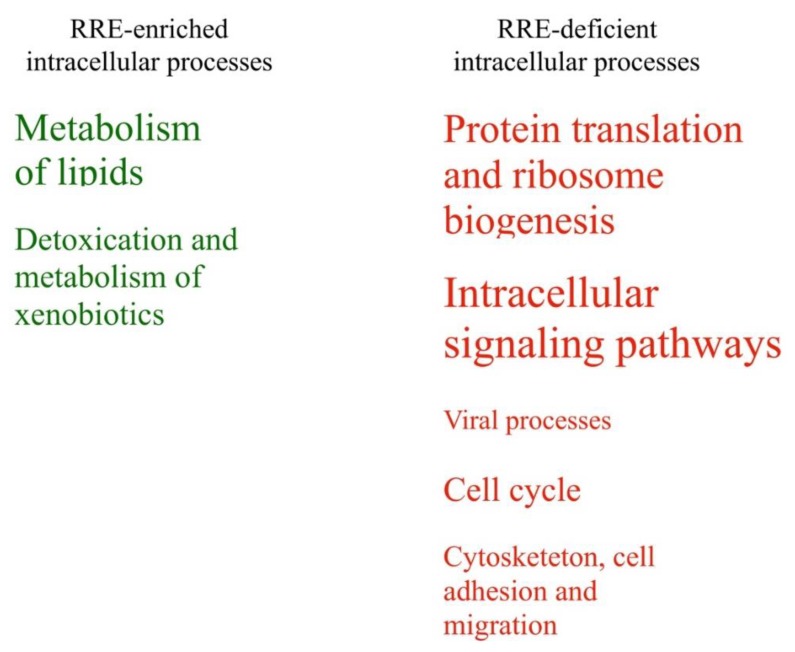
*RRE-enriched* and *RRE-deficient* molecular processes for *evolutionary young* Res.

We compared RRE-enriched and deficient groups of processes identified using Gene Ontology terms and molecular pathway analysis (Table 6, Table 7 and Figure 10). The following *RRE-enriched* processes were identified by evolutionary young REs: (1) metabolism of lipids, (2) detoxication and metabolism of xenobiotics. The processes with the *RRE-deficient* regulation were: (3) cell cycle; (4) cytoskeleton, cell adhesion and migration; (5) protein translation and ribosome biogenesis; (6) intracellular signaling pathways; and (7) processes of viral life cycle. 

The compositions of these groups are identical to those observed for *all* REs, except for a new RRE-deficient group “cytoskeleton, cell adhesion and migration”. It included pathways of cell adhesion, cell-cell junction, cell migration, myoblast fusion, and actin cytoskeleton reorganization. 

All these groups were supported by different numbers of activated pathways and GO terms (Table 6). Schematically, these intracellular processes linked with enriched or deficient RRE-regulation by *evolutionary young* REs and are shown in Figure 10 in relation to the number of features. Twelve other groups of processes (50%) were identified as ambiguously being RRE-enriched or deficient using only one of the methods used (either *gene*- or *pathway*-based), and four groups (~17% of the total amount of groups considered) showed ambiguous trends in both methods (Table 6). Analysis of immunity consensus processes also did not reveal concordant trends by GO and pathway analysis (Table 7).

## 4. Discussion

In this study, for the first time, we combined two independent types of data analysis to identify the molecular processes impacted by the RE-linked TFBS regulation in 13 cell lines representing eight human tissues, on two different evolutionary time scales. The *gene* level analysis was performed by classifying Gene Ontology (GO) annotation features, and the *pathway* level analysis was done by interrogating a high-throughput Oncobox molecular pathway database. These approaches identified different sets of molecular processes being either enriched or deficient in the RE-linked TFBS regulation. Based on RE families’ mean divergence from the respective consensus sequences, we set two different evolutionary time scales for this type of analysis. First, at the level of mammalian radiation (reflected by *all* REs), and second, at the level of radiation of human ancestry and New-World monkeys (reflected by *evolutionary young* REs). Of them, for *all* REs, 13 types of processes (46%) coincided for the two types of analysis and 14 processes (50%) were specific for only one of the above methods used. One process (4%) had contradictory trends at the *gene* and *pathway* levels. The consensually regulated processes were *RRE-enriched*: posttranscriptional silencing by small RNAs, DNA repair, metabolism of amino acids and lipids, detoxication and metabolism of xenobiotics, sensory perception and neurotransmission, fertilization, and T- and NK-cellular immune response. The *RRE-deficient* processes were related to: nucleotide and DNA metabolism, maintenance and modulation of chromatin structure, protein translation and ribosome biogenesis, intracellular signaling pathways, and cellular mechanisms of antiviral response. 

At more recent evolutionary scale, only eight processes (33%) showed congruent results for the two approaches used, whereas most of the processes had ambiguous trends. These results most likely suggest that the method of RRE-analysis used here has different utility for the different evolutionary time scales. For the deeper evolutionary horizon (mammalian radiation), the results were more robust and reproducible than for the fleet horizon (human–New-World monkeys radiation).

The molecular pathways investigated here represent processes that are cumulatively differentially impacted at the level of gene expression regulation by retrotransposons. Retrotransposons are considered pacemakers of eukaryotic genome evolution [9] and were shown to be the major source of transcription factor binding sites (TFBS) for the mammalian DNA [1, 5, 7]. SNPs and nucleotide substitutions mediate continuous but relatively slow changes in the mammalian genetic landscapes [65]. In contrast, insertion of a retrotransposon has potential to dramatically transform the genomic background by immediately providing a totally new sequence of up to 10 kb long, which is non-homologous and non-orthologous to the pre-integration locus as in the case of human REs [66]. These newcomer elements contain numerous functional TFBS that can donate to a new genetic neighborhood, including genes [67]. Moreover, following mutations and epigenetic landmarks can further transform these TFBS profiles, thus enhancing the evolution of gene regulation [24]. A higher proportion of RE-linked TFBS, therefore, can be considered a marker of faster evolution of gene regulation, and vice versa. Our data, therefore, point to the genes and molecular processes that can be regarded as having especially high or especially low rates of evolution in terms of gene expression regulation on different evolutionary time scales. 

In many aspects, our results concerning the regulatory evolution of human molecular pathways are congruent with the previous findings obtained using different methods. For example, the processes of ribosome biogenesis and protein synthesis, as well as nucleotide and DNA metabolism, are highly evolutionary conserved in all domains of life [68,69]. Here, we showed that the regulatory evolution rate of these processes in human lineage is also relatively slow. Contrarily, the regulatory networks of DNA repair evolve quickly according to our results, which is in accord with high redundancy and promiscuity of DNA repair enzymatic systems [70] that need to be tightly regulated especially in long-living organisms to prevent proliferative disorders [71]. We show that the evolution of immune response shows contradictory trends: T- and NK-cellular immune responses (intercellular antiviral response) are evolving rapidly, whereas the mechanisms of intracellular antiviral defense are changing slowly. This pattern is in line with the previous finding that cellular immune response to viruses (especially adaptive T-cell mediated response) is a more recent evolutionary innovation [72]; its regulatory evolution, therefore, can go faster. Similarly, the regulation of amino acids and lipids metabolism also changes relatively quickly, which can relate to changes in nutritional adaptation during evolution [69]. An extremely fast evolutionary signature was observed for the mechanisms of posttranscriptional silencing by small RNAs. This silencing is a primary mechanism repressing newly integrated REs and other intracellular pathogens [73]. Its regulatory networks are permanently evolving, reflecting an intragenome evolutionary arms race between host genes, REs and invasive pathogens. 

Here, we investigated the gene regulatory evolution features at the level of transcription factor binding sites. However, targeted profiling of the additional functional landmarks of the human genome such as covalent histone modifications, DNA methylation and nucleosome positioning, can provide further avenues for studying the regulatory potential and evolutionary impact of transposable elements. Regulation by histone modification can be activating and repressive; therefore, the investigation of RE-driven regulatory evolution for different histone marks could provide a complex and integrative pattern of epigenetic evolution. Another direction of future works could be the comparison of molecular evolution at the level of gene regulation (such as studied in this report) with the evolution of the gene/protein structure. Moreover, these exploratory approaches could be applied to a broad number of model organisms not limited to mammalian or vertebrate species. The feasibility of using the same computational approach in various organisms depends on (i) availability of epigenomic data such as of TFBS and histone modification sites and on (ii) knowledge of molecular pathways in the organisms under investigation. For example, we presume that human molecular pathways can be used only for closely related species such as higher primates. Certain modifications of the pathway contents may expand their utility also on the other mammalian species. For example, we expect that the investigation of mouse regulatory evolution in comparison to humans will be possible only when orthologous genes will be considered. The analysis of more distantly related organisms such as zebrafish, drosophila or Arabidopsis, would, therefore, require revision of molecular pathway databases in addition to complete sets of epigenomic data. 

The current computational approach has an important limitation: its resolution of evolutionary time is relatively low, concerning here only two key points (radiation of New World monkeys and radiation of major eutherian clades). Future research can improve time-precision by considering more evolutionary important events, that can be selected individually for the different species. Comparative analysis of the same evolutionary horizon (such as the origin of Amniotes) for two or more species could provide new insights into our understanding of molecular determinants governing phenotypic macroevolution. Furthermore, the concept of *regulatory evolutionary clock* (or, shortly, regulatory clock) can be tested in the future by comparing scores of RE regulatory load for multiple organisms with established dates of evolutionary divergence. Another limitation of our approach can be the simultaneous interrogation of many different TFs (here we investigate DNA binding patterns of 563 TFs) to calculate one characteristic value for each gene. Further research concerning TFs grouped by tissue specificity and mechanism of action can provide a more detailed view of RE-directed evolution. Finally, we believe that further applications of the analytic approach discovered here for various model organisms will shed light on the general evolution of eukaryotic regulatory networks.

## 5. Conclusions

In this study, we extracted human molecular processes undergoing a relatively fast and slow evolution of their transcriptional regulation under the impact of newly integrating REs. Processes were identified for different evolutionary timescales: (1) radiation of New World monkeys and (2) radiation of major eutherian clades. The most quickly evolving processes were concerning gene regulation by noncoding RNAs including microRNAs, olfaction, color vision, fertilization, T- and NK-cellular immune response, amino acids and fatty acids metabolism, and detoxication. Groups of the relatively slow regulatory evolution were connected with protein biosynthesis, RNA transcription and processing, nuclear chromatin organization, and intracellular molecular signaling. Finally, we propose a quantitative measure of the rate of regulatory evolution that can be calculated reproducibly in different organisms.

## Figures and Tables

**Table 1:** Overall TFBS statistics. Note the significant proportion of round numbers for mapped TFBS because of IDR thresholds used in the standard ENCODE peak called pipeline [46].

Cell Line	Number of TFs Profiled	Number of Mapped TFBS	Number of TFBS Mapped on SINEs	Percentage of TFBS Mapped on SINEs	Number of TFBS Mapped on LINEs	Percentage of TFBS Mapped on LINEs	Number of TFBS Mapped on LR/ERVs	Percentage of TFBS Mapped on LR/ERVs
**K562**	265	78,021,500	25,078,428	32.1	22,646,141	29	10,394,662	13.3
**HepG2**	175	51,982,065	16,406,062	31.6	140,104,77	27	6,493,562	12.5
**HEK293**	177	53,100,000	19,214,015	36.2	15,708,687	29.6	6,089,813	11.5
**GM12878**	127	37,688,353	10,185,415	27	9,927,943	26.3	4,727,719	12.5
**MCF-7**	80	23,851,396	7,039,271	29.5	7,499,703	31.4	3,297,443	13.8
**A549**	44	13,044,409	3,930,407	30.1	3,569,214	27.4	1,667,464	12.8
**HeLa-S3**	15	4,500,000	1,112,669	24.7	1,036,667	23	566,618	12.6
**SK-N-SH**	15	4,500,000	874,572	19.4	958,401	21.3	473,342	10.5
**HCT116**	4	1,200,000	402,872	33.6	333,771	27.8	181,562	15.1
**Ishikawa**	4	1,200,000	268,209	22.4	266,191	22.2	141,530	11.8
**HEK293T**	17	5,100,000	129,7870	25.4	1,725,589	33.8	618,745	12.1
**MCF_10A**	3	900,000	214,458	23.8	215,576	24	103,925	11.5
**GM12891**	7	2,100,000	559,260	26.6	433,194	20.6	253,577	12.1

**Table 2:** Gene neighborhood-linked TFBS statistics.

Cell Line	Number of TFs Profiled	Number of Mapped TFBS	Number of TFBS Mapped on SINEs	Percentage of TFBS Mapped on SINEs	Number of TFBS Mapped on LINEs	Percentage of TFBS Mapped on LINEs	Number of TFBS Mapped on LR/ERVs	Percentage of TFBS Mapped on LR/ERVs
**K562**	260	12,547,055	4,667,810	37.2	2,508,956	20	1,081,084	8.6
**HepG2**	175	8,803,748	3,023,414	34.3	1,559,929	17.7	669,907	7.6
**HEK293**	177	8,074,128	3,235,573	40.1	1,569,002	19.4	587,798	7.3
**GM12878**	127	5,626,411	1,699,882	30.2	970,626	17.3	400,502	7.1
**MCF-7**	80	3,067,441	1,029,118	33.5	598,982	19.5	238,005	7.8
**A549**	44	2,035,359	662,458	32.5	358,084	17.6	142,842	7
**HeLa-S3**	15	720,963	193,365	26.8	109,683	15.2	47,759	6.6
**SK-N-SH**	15	679,254	139,230	20.5	90,313	13.3	36,751	5.4
**HCT116**	4	202,223	71,590	35.4	38,293	18.9	16,878	8.3
**Ishikawa**	4	194,053	52,211	26.9	28,867	14.9	12,768	6.6
**HEK293T**	17	518,287	162,440	31.3	115,555	22.3	42,242	8.2
**MCF_10A**	3	145,892	43,930	30.1	25,500	17.5	10,304	7.1
**GM12891**	7	427,212	94,464	22.1	48,947	11.5	21,515	5

**Table 3:** RRE-enriched and deficient intracellular processes according to Gene Ontology (GO) and molecular pathway analysis (*all* REs).

ID	Group of Processes	RRE Enrichment by Pathway Analysis		RRE Enrichment by GO Analysis		Overall Status
		Enriched pws	Deficient pws	Enriched GO terms	Deficient GO-terms	
1	Posttranscriptional silencing by small RNAs	1	0	1	0	RRE enriched
2	DNA repair	2	0	5	0	RRE enriched
3	Amino acids, Peptides and Polyamines Metabolism	20	5	13	8	RRE enriched
4	Lipid Metabolism	14	7	11	0	RRE enriched
5	Detoxication, Metabolism of Xenobiotics and Rare Molecules	13	0	4	0	RRE enriched
6	Sensory Perception and Neurotransmission	7	0	10	0	RRE enriched
7	Fertilization	1	0	9	0	RRE enriched
8	Cellular Immune Response (T cells and NK cells)	11	0	7	6	RRE enriched
9	Nucleic Base, Nucleosides and Nucleotides Metabolism	6	9	0	24	RRE deficient
10	DNA metabolism and Chromatin structure	0	4	0	151	RRE deficient
11	Translation and Protein Quality Control	0	12	8	130	RRE deficient
12	Intracellular Signaling	22	94	5	48	RRE deficient
13	Response to Viruses	0	3	0	17	RRE deficient
14	Vitamin Metabolism	4	0	0	0	RRE enriched
15	Hormones	6	0	0	0	RRE enriched
16	Molecular Transport	10	0	0	0	RRE enriched
17	Sulfur Metabolism and Linked Redox Reactions	5	0	0	0	RRE enriched
18	Metal Metabolism	0	0	6	0	RRE enriched
19	Response to Phorbol Acetate	0	0	0	3	RRE deficient
20	Electron Transfer Reactions	0	0	5	17	RRE deficient
21	Mitochondria	0	0	5	17	RRE deficient
22	RNA Synthesis and Degradation	0	0	0	139	RRE deficient
23	Cell Adhesion and Interaction	0	0	0	15	RRE deficient
24	Cell Cycle and Mitosis	0	0	0	55	RRE deficient
25	Cell Death	0	0	0	41	RRE deficient
26	Protein Localization and Modification	0	0	0	19	RRE deficient
27	Response to Physical and Chemical Stress	0	0	0	24	RRE deficient
28	Carbohydrates Metabolism	5	3	0	9	Ambiguous Pattern
29	Immunity	36	16	23	45	Shown separately
30	Other/Too General Terms	0	0	13	17	N/A

**Table 4:** RRE-enriched and deficient immunity-linked processes according to Gene Ontology (GO) and molecular pathway analysis (*all* REs).

Group of Processes	RRE Enrichment by Pathway Analysis		RRE Enrichment by GO Analysis		Overall Status
	Enriched pws	Deficient pws	Enriched GO terms	Deficient GO-terms	
Autoimmunity	4	0	0	0	RRE enriched
Blood Clotting	2	0	0	0	RRE enriched
Innate Immunity	8	0	0	5	Ambiguous
Inflammation	3	5	0	0	Ambiguous
Cellular Immune Response (T cells and NK cells)	11	0	7	6	RRE enriched
Activation of Antigen-Presenting Cells by T-helper cells	2	7	0	0	RRE deficient
Other/Too General Terms	6	1	8	11	Ambiguous
Immune Cells Migration and Activation	0	0	7	0	RRE enriched
Activity and maturation of B cells	0	0	0	6	RRE deficient

**Table 5:** RRE-enriched and deficient microRNA and lncRNA genes (*all* REs).

**Gene set**	**MiRs**	**Genes, totally**	**Hypergeometric p-value**	**Hypothesis tested**
RRE-enriched	177	1219	2.416 × 10^−18^	miRNA are enriched
RRE-deficient	72	1219	0.0138	miRNA are not enriched
Totally—1865 miRNA genes in 25,075 genes of the human genome				
**Gene set**	**lncRNAs**	**Genes, totally**	**Hypergeometric p-value**	**Hypothesis tested**
RRE-enriched	150	1219	1.9500× 10^−17^	lncRNA are enriched
RRE-deficient	18	1219	2.42× 10^−16^	lncRNA are not enriched
Totally—1505 lncRNA genes in 25,075 genes of the human genome				

**Table 6:** RRE-enriched and deficient intracellular processes according to Gene Ontology (GO) and molecular pathway analysis (*evolutionary young* REs).

ID	Group of Processes	RRE Enrichment by Pathway Analysis		RRE Enrichment by GO Analysis		Overall Status
		Enriched pws	Deficient pws	Enriched GO terms	Deficient GO-Terms	
1	Lipids metabolism	22	8	2	0	RRE enriched
2	Signaling	28	40	8	31	RRE deficient
3	Immune System	18	14	3	12	Shown Separately
4	Cell cycle	1	6	0	62	RRE deficient
5	Cell death	7	6	0	35	Ambiguous Pattern
6	Amino acids and polyamines metabolism	13	5	0	0	RRE enriched
7	Metabolism and detoxication of xenobiotics	8	0	2	0	RRE enriched
8	Sulfur-linked reactions	6	0	0	0	RRE enriched
9	Vitamins metabolism	10	0	0	0	RRE enriched
10	Carbohydrates and related molecules metabolism	9	5	0	6	Ambiguous Pattern
11	Nucleic base, nucleotides and nucleosides metabolism	5	3	0	14	Ambiguous Pattern
12	Transport of small molecules	4	0	0	0	RRE enriched
13	Blood Clotting	3	0	0	0	RRE enriched
14	Cytosketeton, cell adhesion and migration	0	18	0	16	RRE deficient
15	Endocytosis	0	4	0	0	RRE deficient
16	Translation and protein quality control	0	23	0	105	RRE deficient
17	Viruses	0	7	0	18	RRE deficient
18	Signal perception and neurotransmission	0	0	22	0	RRE enriched
19	RNA Synthesis and Degradation	0	0	0	80	RRE deficient
20	DNA metabolism and chromatin	0	0	0	66	RRE deficient
21	Protein Localization and Modification	0	0	0	20	RRE deficient
22	Response to Physical and Chemical Stress	0	0	0	15	RRE deficient
23	Oxidative Phosphorylation in Mitochondria	0	0	0	19	RRE deficient
24	Other/Too General Terms	18	12	18	231	N/A

**Table 7:** RRE-enriched and deficient immunity-linked processes according to Gene Ontology (GO) and molecular pathway analysis (*evolutionary young* REs).

Group of Processes	RRE enrichment by pathway analysis		RRE enrichment by GO analysis		Overall status
	Enriched pws	Deficient pws	Enriched GO terms	Deficient GO-terms	
Innate immunity	5	2	0	9	Ambiguous Pattern
Inflammation	3	3	0	0	Ambiguous Pattern
T-cells mediated immunity	4	3	0	0	RRE enriched
Other/Too General Terms	5	0	3	3	

## Supplementary Materials

The following are available online at http://www.mdpi.com/2073-4409/8/2/130/s1.

Supplementary file 1—mathematical description of proposed calculation of RE-linked TFBS regulatory impact on human genes and molecular pathways.

Supplementary file 2—the list of transcription factors investigated here and raw ENCODE data files for each cell line.

Supplementary file 3—pairwise correlation for GRE and NGRE and numbers of transcription factors investigated in each cell line.

Supplementary file 4—pairwise correlation for PII and NPII and numbers of transcription factors investigated in each cell line.

Supplementary file 5—overall data analysis pipeline used in current study.

Supplementary file 6—the distribution of TFBS hits for the different transcriptional factor proteins varied among the different cell lines. Color scale shows depth of TFBS mapping; blank spaces mean absence of TFBS data for the respective transcriptional factor in the context of a given cell line in ENCODE dataset.

Supplementary file 7—the gene RE-linked TFBS absolute and normalized enrichment scores *GRE* and *NGRE*, respectively.

Supplementary file 8—the molecular pathway RE-linked TFBS absolute and normalized enrichment scores *PII* and *NPII*, respectively.

Supplementary file 9—RRE-enriched and RRE-deficient genes.

Supplementary file 10—manually classified RRE-enriched and RRE-deficient molecular pathways for *all* REs.

Supplementary file 11—manually classified RRE-enriched and RRE-deficient GO terms for *all* REs.

Supplementary file 12—manually classified RRE-enriched and RRE-deficient GO terms for *evolutionary young* REs.

Supplementary file 13—manually classified RRE-enriched and RRE-deficient molecular pathways for *evolutionary young* REs.
