# Correction: Nikitin, D., et al. Retroelement—Linked Transcription Factor Binding Patterns Point to Quickly Developing Molecular Pathways in Human Evolution. *Cells* 2019, *8*, 130

**DOI:** 10.3390/cells8080832

**Published:** 2019-08-05

**Authors:** Daniil Nikitin, Andrew Garazha, Maxim Sorokin, Dmitry Penzar, Victor Tkachev, Alexander Markov, Nurshat Gaifullin, Pieter Borger, Alexander Poltorak, Anton Buzdin

**Affiliations:** 1 I.M. Sechenov First Moscow State Medical University, 119992 Moscow, Russia; 2 Omicsway Corp., Walnut, CA 91798, USA; 3 D. Rogachev Federal Research Center of Pediatric Hematology, Oncology and Immunology, 117198 Moscow, Russia; 4 Shemyakin-Ovchinnikov Institute of Bioorganic Chemistry, 117198 Moscow, Russia; 5 Faculty of Bioengineering and Bioinformatics, Lomonosov Moscow State University, 119992 Moscow, Russia; 6 Faculty of Biology, Moscow State University, 119192 Moscow, Russia; 7 Faculty of Fundamental Medicine, Moscow State University, 119992 Moscow, Russia; 8 Laboratory of the Swiss Hepato-Pancreato-Biliary (HPB) and Transplantation Center, Department of Surgery, University Hospital Zürich, Raemistrasse 100, Zürich CH-8091, Switzerland; 9 Program in Immunology, Sackler Graduate School, Tufts University, Boston, MA 02111, USA

In the article ‘Retroelement—Linked Transcription Factor Binding Patterns Point to Quickly Developing Molecular Pathways in Human Evolution,’ a number of transcription factor binding sites (TFBS) mapped on all retroelement classes were incorrectly calculated as sum of TFBS numbers separately mapped on LINEs, SINEs and LTR retrotransposons/endogenous retroviruses (LR/ERVs). This made the proportion of RE-linked TFBS 72.1% (incorrect) instead of 55.5% (correct) of all mapped TFBS. Similarly, the proportion of RE-linked TFBS was incorrectly calculated as 61.4% (incorrect) instead of 47% (correct) of all TFBS mapped in 10 kb gene transcriptional start neighborhood. Corrections were made in the text and Table 1 and Table 2.

For example, in Table 1, the total TFBS numbers mapped on SINEs, LINEs and LTRs in all 13 cell lines tested were 86,583,508, 78,331,554 and 3,009,962, respectively. The total number of mapped TFBS in 13 cell lines was 277,187,723. The number of TFBS mapped on all retroelements classes (LINEs, SINEs and LR/ERVs) was incorrectly calculated as the simple arithmetic sum of these three numbers, i.e., 199,925,024, or 72.1% of all mapped TFBS. Similarly, a proportion of gene neighboring TFBS was calculated as 61.4% or all TFBS. This method of calculation was incorrect because it did not take into account that one TFBS could simultaneously overlap with several individual LINE, SINE or LR/ERV elements. 

Here, we made corrections by directly mapping TFBS on all retroelement classes. The corrected TFBS numbers are shown on Table 1.

Table 1 should read as follows: 

Similarly, we re-calculated corrected the number and percentage of TFBS mapped on all retroelement classes in 10 kb gene neighborhoods (Table 2).

Table 2 should read as follows: 

The passage on the page 7 between the Table 1 and Table 2 should read as follows:

“In total, 277,187,723 TFBS hits could be mapped on the human genome for all these cell lines. Of them, 55.5% overlapped with the RE sequences, thus confirming that REs serve as the major source of TFBS in human cells. Considering previous reports, this proportion may seem high [60]. However, in our analysis, all multimapped TFBS reads were filtered out according to the standard ENCODE ChIP-seq mapping and filtering pipeline [42], so the results represented uniquely mapped TFBS reads. To confirm this TFBS proportion, we also validated the method used by parallel mapping of all human RE sequences extracted from USCS Genome Browser [55] on the human genome. In a good agreement with previously published data [1], REs mapped by the same approach occupied ~45% of human DNA, genome assembly hg19. We therefore found no technological drawbacks here and suggest that the proportion of 55.5% RE-linked TFBS is correct at least for the ENCODE primary cell culture datasets used. However, this proportion was somewhat lower for the TFBS mapped in a 10 kb neighborhood of gene transcriptional start sites (TSS): Among 43,042,026 totally mapped TFBS, only 47% overlapped with REs. This overall trend was representative for all cell lines under investigation (Table 2). The TSS-proximal TFBS hits were unevenly distributed among the major classes of REs: ~30% were attributed to SINEs; ~17%–to LINEs, and ~7%–to LTR retrotransposons and endogenous retroviruses. For the total fraction of RE-linked TFBS hits (not only gene-proximal), these proportions were, respectively, 28%, 26% and 12%.”

The beginning of the last paragraph in “Background” section should read as follows:

“To distinguish cell type-specific and general evolutionary features, in this study, we investigated the distributions of RE-linked hits for all 13 human cell lines TFBS-profiled for 563 DNA-binding proteins during the ENCODE project [40], representing eight different tissues/organs from the different individuals (Supplementary file 2, Figure S2A). We found that 55.5% of totally mapped TFBS overlapped with the RE sequences, thus confirming RE’s status as the major source of TFBS for human cells.”

## Figures and Tables

**Table 1:** Overall transcription factor binding sites (TFBS) statistics.

Cell Line	Number of TFs Profiled	Number of Mapped TFBS	Number of TFBS Mapped on SINEs	Percentage of TFBS Mapped on SINEs	Number of TFBS Mapped on LINEs	Percentage of TFBS Mapped on LINEs	Number of TFBS Mapped on LR/ERVs	Percentage of TFBS Mapped on LR/ERVs	Number of TFBS Mapped on All Classes	Percentage of TFBS Mapped on All Classes
K562	265	78,021,500	25,078,428	32.1	22,646,141	29	10,394,662	13.3	47,072,030	60.3
HepG2	175	51,982,065	16,406,062	31.6	140,104,77	27	6,493,562	12.5	30,359,142	58.4
HEK293	177	53,100,000	19,214,015	36.2	15,708,687	29.6	6,089,813	11.5	33,394,943	62.9
GM12878	127	37,688,353	10,185,415	27	9,927,943	26.3	4,727,719	12.5	20,728,922	55
MCF-7	80	23,851,396	7,039,271	29.5	7,499,703	31.4	3,297,443	13.8	14,788,900	62
A549	44	13,044,409	3,930,407	30.1	3,569,214	27.4	1,667,464	12.8	7,524,691	57.7
HeLa-S3	15	4,500,000	1,112,669	24.7	1,036,667	23	566,618	12.6	2,311,571	51.3
SK-N-SH	15	4,500,000	874,572	19.4	958,401	21.3	473,342	10.5	2,015,301	44.3
HCT116	4	1,200,000	402,872	33.6	333,771	27.8	181,562	15.1	732,432	61
Ishikawa	4	1,200,000	268,209	22.4	266,191	22.2	141,530	11.8	588,478	49
HEK293T	17	5,100,000	129,7870	25.4	1,725,589	33.8	618,745	12.1	3,050,402	59.8
MCF_10A	3	900,000	214,458	23.8	215,576	24	103,925	11.5	449,290	49.9
GM12891	7	2,100,000	559,260	26.6	433,194	20.6	253,577	12.1	1,058,657	50.4

**Table 2:** Gene neighborhood-linked TFBS statistics.

Cell Line	Number of TFs Profiled	Number of Mapped TFBS	Number of TFBS Mapped on SINEs	Percentage of TFBS Mapped on SINEs	Number of TFBS Mapped on LINEs	Percentage of TFBS Mapped on LINEs	Number of TFBS Mapped on LR/ERVs	Percentage of TFBS Mapped on LR/ERVs	Number of TFBS Mapped on All Classes	Percentage of TFBS Mapped on All Classes
**K562**	260	12,547,055	4,667,810	37.2	2,508,956	20	1,081,084	8.6	6,782,652	54.1
**HepG2**	175	8,803,748	3,023,414	34.3	1,559,929	17.7	669,907	7.6	4,366,650	49.6
**HEK293**	177	8,074,128	3,235,573	40.1	1,569,002	19.4	587,798	7.3	4,450,113	55.1
**GM12878**	127	5,626,411	1,699,882	30.2	970,626	17.3	400,502	7.1	2,589,335	46
**MCF-7**	80	3,067,441	1,029,118	33.5	598,982	19.5	238,005	7.8	1,560,667	50.9
**A549**	44	2,035,359	662,458	32.5	358,084	17.6	142,842	7	969,614	47.6
**HeLa-S3**	15	720,963	193,365	26.8	109,683	15.2	47,759	6.6	301,213	41.8
**SK-N-SH**	15	679,254	139,230	20.5	90,313	13.3	36,751	5.4	233,827	34.4
**HCT116**	4	202,223	71,590	35.4	38,293	18.9	16,878	8.3	103,766	51.3
**Ishikawa**	4	194,053	52,211	26.9	28,867	14.9	12,768	6.6	80,822	41.6
**HEK293T**	17	518,287	162,440	31.3	115,555	22.3	42,242	8.2	266,806	51.5
**MCF_10A**	3	145,892	43,930	30.1	25,500	17.5	10,304	7.1	66,090	45.3
**GM12891**	7	427,212	94,464	22.1	48,947	11.5	21,515	5	179,956	42.1
