# Supplementary material for: Retroelement—Linked Transcription Factor Binding Patterns Point to Quickly Developing Molecular Pathways in Human Evolution
Source: Cells. 2019 Feb 6;8(2):130. doi: 10.3390/cells8020130 (PMC6406739; doi:10.3390/cells8020130)
Supplement: Supplementary file 1 [file cells-08-00130-s001.zip › Supplementary5.pdf]

- **Quantization of RE-linked TFBS impacts on genes and molecular pathways**

GRE and NGRE scores (gene level of data analysis)

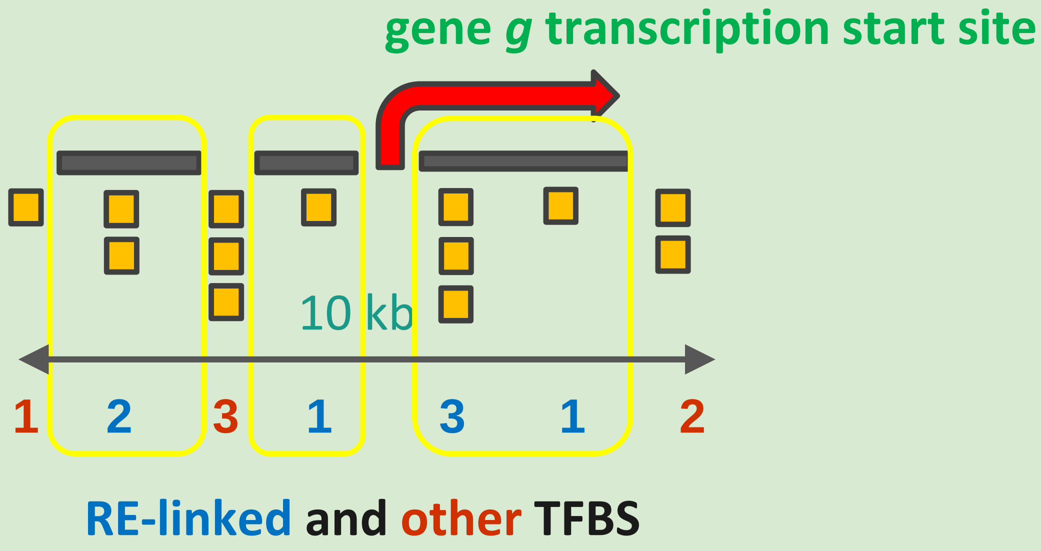

$TESg = \sum$ TFBS in REs in gene $g = 7$

$TTSg = \sum$ TFBS + TFBS in gene $g = 7 + 6$

Gene RE-linked TFBS Enrichment (GRE)

$$GREg = \frac{TESg}{TES_m}$$ (TES for gene g) (mean TES for all genes)

$$GTEg = \frac{TTSg}{TTSm}$$ (TTS for gene g) (mean TTS for all genes)

Normalized GRE (NGRE)

$$NGREg = \frac{GREg}{GTEg}$$ (GRE for gene g) (GTE for gene g)

High *GRE* = High *number* of RE-linked TFBS in a gene

High *NGRE* = High *proportion* of RE-linked TFBS in a gene

PII and NPII scores (pathway level of data analysis)

Pathway Involvement Index (PII)
Normalized Pathway Involvement Index (NPII)

$$PIIp = \frac{\sum_{i=1}^{n} GREi}{n}$$

(sum of GRE for all pathway genes)

(number of genes in a pathway)

$$PGIp = \frac{\sum_{i=1}^{n} GTEi}{n}$$

(sum of GTE for all pathway genes)

(number of genes in a pathway)

Normalized PII (NPII)

$$NPIIp = \frac{PIIp}{PGIp}$$

(PII for pathway *p*)

(PGI for pathway *p*)

High *PII* = High *number* of RE-linked TFBS for pathway components

High *NPII* = High *proportion* of RE-linked TFBS for pathway components

Data analysis pipeline

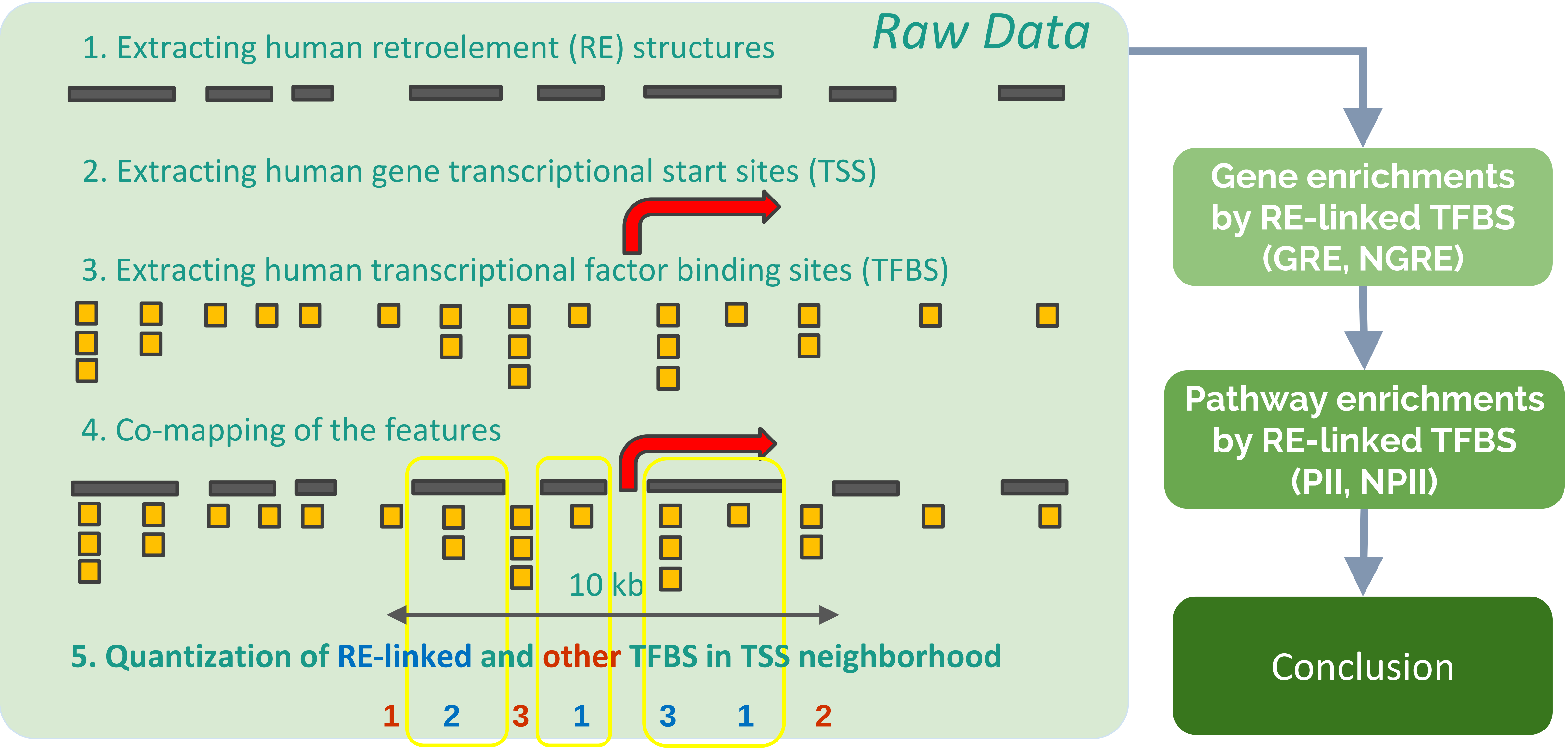

Data analysis platform

Structure Databank

✓ 3.124 molecular pathways for >8.000 connected human genes

✓ >24.000 human gene structures

Experimental Database

✓ ENCODE project experimental data

✓ Transcription Factor Binding Site (TFBS) profiles for 563 transcription factor proteins

✓ Totally ~21 million human TFBS

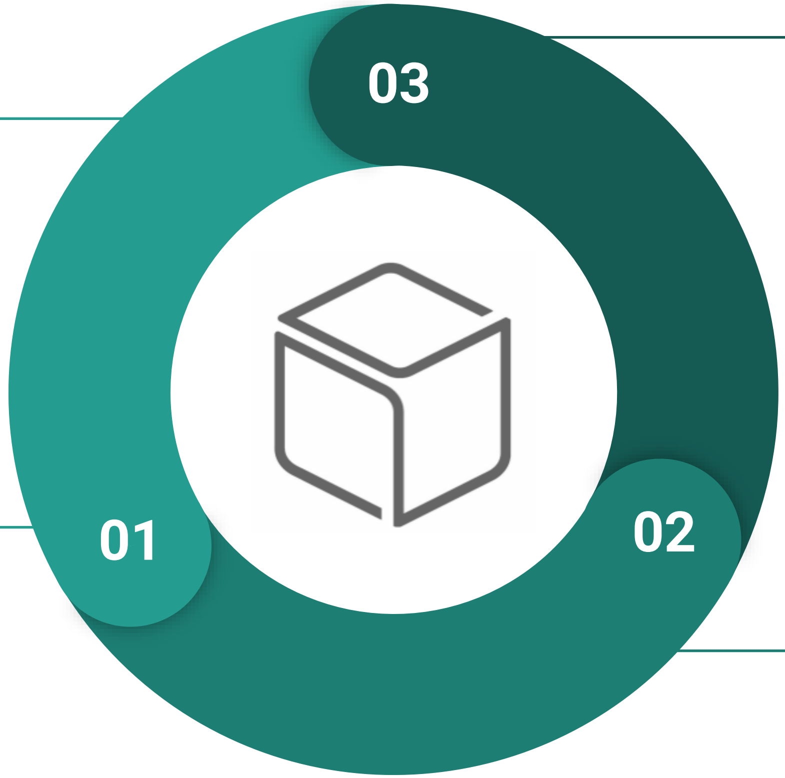

Analytic Algorithm

✓ Calculation of GRE scores for gene transcriptional start neighborhood enrichments by RE-linked TFBS

✓ Calculation of NGRE scores for *normalized* gene enrichments

✓ Calculation of PII scores for pathway-aggregated enrichments by RE-linked TFBS

✓ Calculation of NPII scores for *normalized* pathway enrichments

Features Mapping

✓ Mapping all gene transcriptional start 10-kb neighborhoods

✓ Mapping all human retrotransposons

✓ Mapping all human TFBS
