# Supplementary figures and images for: Retroelement—Linked Transcription Factor Binding Patterns Point to Quickly Developing Molecular Pathways in Human Evolution
Source: Cells. 2019 Feb 6;8(2):130. doi: 10.3390/cells8020130 (PMC6406739; doi:10.3390/cells8020130)

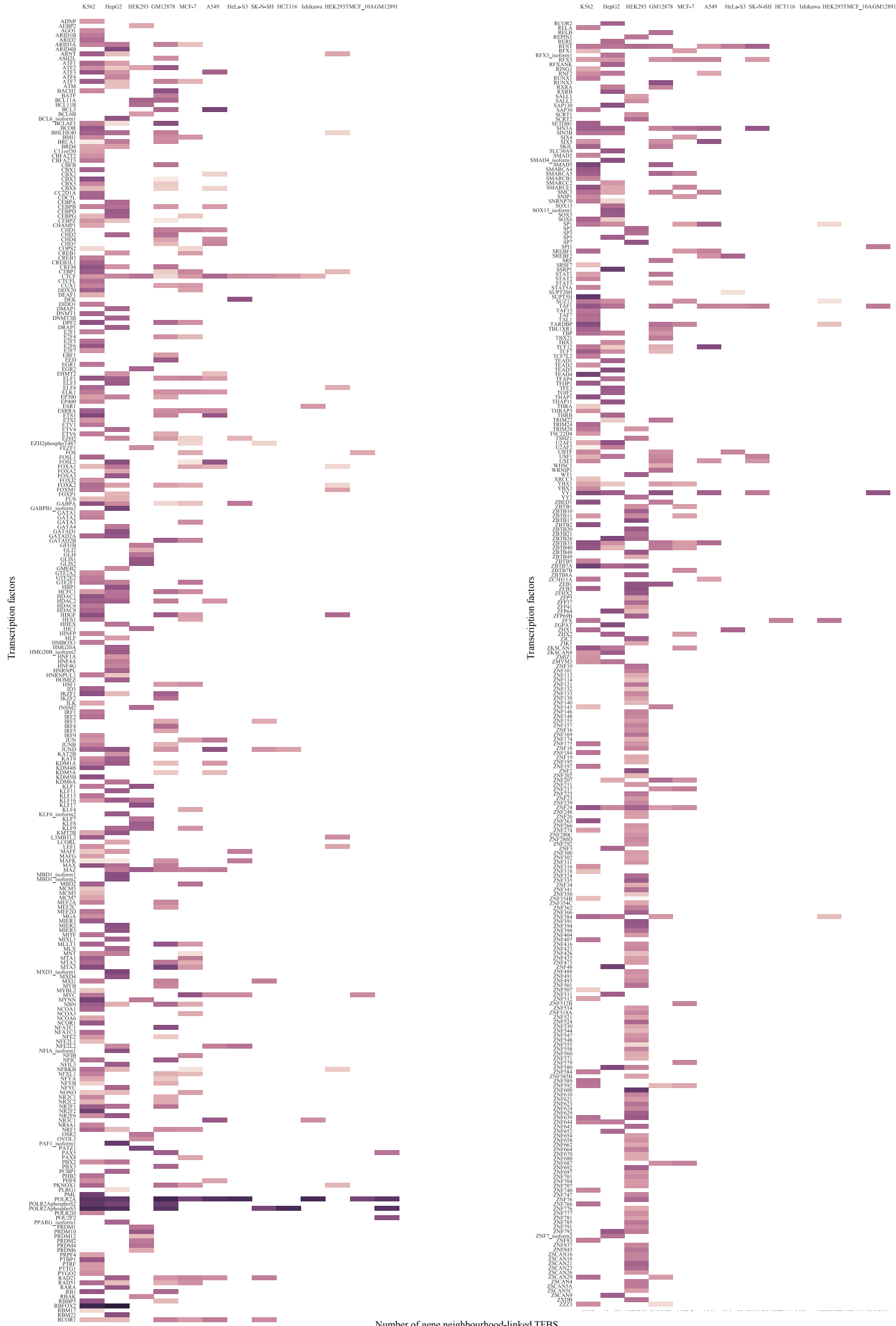

Supplement: Supplementary file 1 [file cells-08-00130-s001.zip › Supplementary6.pdf]
